# Anomalous Hall magnetoresistance in a ferromagnet

**DOI:** 10.1038/s41467-018-04712-9

**Published:** 2018-06-08

**Authors:** Yumeng Yang, Ziyan Luo, Haijun Wu, Yanjun Xu, Run-Wei Li, Stephen J. Pennycook, Shufeng Zhang, Yihong Wu

**Affiliations:** 1 Department of Electrical and Computer Engineering, National University of Singapore, 4 Engineering Drive 3, Singapore, 117583 Singapore; 2 Department of Materials Science & Engineering, National University of Singapore, Singapore, 117575 Singapore; 3 Key Laboratory of Magnetic Materials and Devices, Ningbo Institute of Materials Technology and Engineering, Chinese Academy of Sciences, 315201 Ningbo, Republic of China; 4 Department of Physics, University of Arizona, Tucson, AZ 85721 USA

## Abstract

The anomalous Hall effect, observed in conducting ferromagnets with broken time-reversal symmetry, offers the possibility to couple spin and orbital degrees of freedom of electrons in ferromagnets. In addition to charge, the anomalous Hall effect also leads to spin accumulation at the surfaces perpendicular to both the current and magnetization direction. Here, we experimentally demonstrate that the spin accumulation, subsequent spin backflow, and spin–charge conversion can give rise to a different type of spin current-related spin current related magnetoresistance, dubbed here as the anomalous Hall magnetoresistance, which has the same angular dependence as the recently discovered spin Hall magnetoresistance. The anomalous Hall magnetoresistance is observed in four types of samples: co-sputtered (Fe_1−*x*_Mn_*x*_)_0.6_Pt_0.4_, Fe_1−*x*_Mn_*x*_/Pt multilayer, Fe_1−*x*_Mn_*x*_ with *x* = 0.17–0.65 and Fe, and analyzed using the drift-diffusion model. Our results provide an alternative route to study charge–spin conversion in ferromagnets and to exploit it for potential spintronic applications.

## Abstract (web summary)

Magnetoresistance in ferromagnetic materials and heterostructures have been enabling advanced understanding of electron transport in solids, as well as new concepts for applications. Here the authors demonstrate a different type of magnetoresistance arising from anomalous Hall effect associated spin–charge mutual conversion.

## Introduction

Magnetoresistance (MR) in ferromagnetic (FM) materials and related heterostructures plays essential roles both in fundamental understanding of magnetism and electron transport in these structures and in various technological applications^1–4^. The most widely studied MR effects include anisotropic magnetoresistance (AMR), giant magnetoresistance (GMR) and tunnel magnetoresistance (TMR). These MR effects typically arise from spin-dependent transport of charge carriers either in the bulk or at the interfaces of these structures or the combination of both. Recently, the discovery of several types of MR effects of different origins have triggered a renewed interest for spin-dependent MR; these include spin Hall magnetoresistance (SMR) in FM/heavy metal (HM) bilayers^5–10^, Rashba–Edelstein magnetoresistance (REMR) in Bi/Ag/CoFeB^11^, and Hanle magnetoresistance (HMR) in HMs^12^. One key aspect of these recently discovered MR effects is that they all originate from a two-step charge–spin conversion process, i.e., in the first step charge current is converted to spin current through either the spin Hall effect (SHE)^13, 14^ or the Rashba–Edelstein effect (REE)^15, 16^, and in the second step part of the reflected spin current is converted back to charge current by the respective inverse effects. As inverse SHE (ISHE) always co-exists inside a material with SHE, the interplay of these two gives rise to an extra positive resistance contribution to a bulk conductor, which was first reported in the context of Hall effect in semiconductors^17^. In the proximity of surfaces/edges, part of the SHE generated spin current is canceled out by the reflected spin current due to spin accumulation, resulting in a negative resistance contribution, as first derived by Dyakonov^18^. In the recently observed MR effects, the positive contribution does not play a role because it is insensitive to external field, while the negative contribution is modulated through controlling the amount of spin current reflection by either an adjacent magnetization (SMR and REMR) or an external magnetic field (HMR). Despite their small magnitude, these MRs are powerful tools to extract spin transport parameters, particularly spin–orbit torque (SOT) in FM/HM heterostructures^19–27^, which has important applications in three-terminal^20^, logic^28^, and sensing^29, 30^ devices. Unlike conventional MR effect, in all these MR effects, the FM plays a relatively less important role as both charge to spin and spin to charge conversions take place inside the HM layer. The FM only influences the conversion process indirectly through regulating the amount of spin current reflected back to FM/HM or FM/non-magnetic metal (NM) interfaces. From the application viewpoint, however, it will be of interest to investigate if a MR effect similar to SMR can be present in a FM alone, as this would allow additional flexibility in manipulating the charge–spin conversion process via controlling the magnetization of the FM directly.

Recently, anomalous Hall effect (AHE) in FM has attracted attention as an alternative mechanism for generating spin current or SOT in FM/NM multilayers. When a charge current **j**_**c**_ flows in an FM in the longitudinal direction, spin-up and spin-down electrons are deflected to opposite transverse directions via extrinsic mechanisms like skew scattering and side-jump or intrinsic mechanism related to band structure of the material^31^. Due to the asymmetry in density of states at the Fermi level and charge transport in FM, both transverse charge and spin accumulations will occur at boundaries of the sample at steady state. The former acts on the entire sample, generating the AHE voltage; while the latter leads to a backflow of spin current that only affects the vicinity of the sample boundary. Taniguchi et al.^32, 33^ have predicted theoretically the presence of AHE-related SOT in FM/NM/FM trilayers and MR in FM/NM bilayers. Very recently, several experimental attempts have been made to detect the AHE-induced spin current through either spin injection experiment in Y_3_Fe_5_O_12_/Py heterostructure^34, 35^ or characterization of SOT in FM/NM/FM sandwich structures^36, 37^. However, since all these experiments involve multiple layers, it is difficult to rule out completely contributions other than AHE to the predicted or observed MR or SOT. In this regard, here we report on a MR induced by AHE and its inverse effect in a single FM layer, and refer it to as anomalous Hall magnetoresistance (AHMR). In order to observe the AHMR, one requires FM with a large AHE. Therefore, we focus on four types of FMs, i.e., co-sputtered (Fe_1−*x*_Mn_*x*_)_0.6_Pt_0.4_, Fe_1−*x*_Mn_*x*_/Pt multilayers^38, 39^, Fe_1−*x*_Mn_*x*_ with *x* = 0.17–0.65 and Fe. These materials are chosen because they allow to tune the saturation magnetization and thus the strength of AHE by simply adjusting the Mn composition (except for Fe). Fe_1−*x*_Mn_*x*_ itself can be tuned from ferromagnet to antiferromagnet by controlling the Mn composition. The inclusion of Pt further enhances the AHE in both the co-sputtered and multilayer samples. Magnetoresistance with SMR-like angular dependence is observed in all the four types of samples. We argue that the observed MR is AHMR instead of SMR because all the samples behave as a single phase FM. Our argument is further substantiated by scaling analysis of the AHE and the relation between the MR and anomalous Hall angle. Based on the drift–diffusion formalism, we derive the analytical equations for MR in a single FM layer including AHE, and demonstrate that both the magnitude and thickness dependence of AHMR can be accounted for reasonably well using the analytical model.

## Results

### Angle-dependent MR

**Fig. 1:**
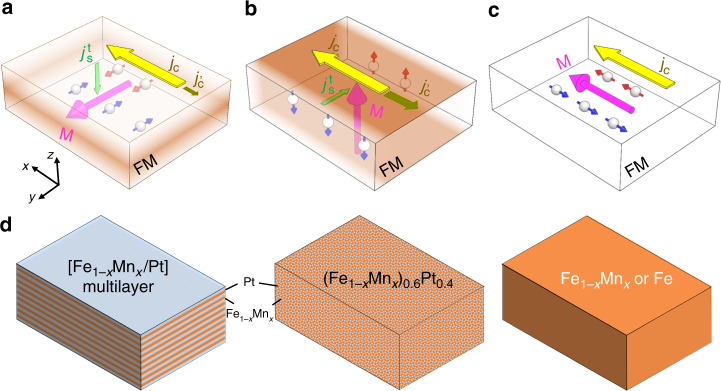
Illustration of AHE and inverse AHE in thin FM films and sample structures used in this study. AHE and inverse AHE in different magnetization configurations: **a**, **m**||**y**; **b**, **m**||**z**; and **c**, **m**||**x**. **d** Schematics of three types of samples including co-sputtered (Fe_1−*x*_Mn_*x*_)_0.6_Pt_0.4_, Fe_1−*x*_Mn_*x*_/Pt multilayer, Fe_1-*x*_Mn_*x*_ with *x* = 0.17–0.65 and Fe. The colormap in **a**–**c** is the distribution of net $j_{\mathrm{s}}^{\mathrm{t}}$: the deeper the color the larger the net spin current

As depicted in Fig. 1a, b the applied charge current (*j*_c_) in *x*-direction induces a transverse spin current ($j_{\mathrm{s}}^{\mathrm{t}}$) via AHE with the flow direction given by **m** × **j**_**c**_, where **m** is the magnetization direction. The simultaneous action of inverse AHE will convert a portion of $j_{\mathrm{s}}^{\mathrm{t}}$ back to charge current ($j_{\mathrm{c}}^\prime$) that has a direction opposite to the original one, thereby increasing the overall resistance of FM (positive contribution). For the case wherein **m**||**y**, with the comparable scale of film thickness and spin diffusion length, the backflow of spin current largely cancels $j_{\mathrm{s}}^{\mathrm{t}}$ and reduces the extra resistance (negative contribution). Whereas, when **m**||**z**, with the large lateral size, such cancellation is confined in the proximity of the sample edges only, and $j_{\mathrm{s}}^{\mathrm{t}}$ inside the sample remains nearly constant. To illustrate the difference in two cases, we illustrate in Fig. 1a, b the distribution of net $j_{\mathrm{s}}^{\mathrm{t}}$ in a colormap, the deeper the color the larger the net spin current. On the other hand, in the case of **m**||**x** (see Fig. 1c), there is no AHE. Therefore, when the magnetization rotates in the *yz*-plane, an angle-dependent MR, i.e., AHMR, appears and its dependence is expected to be the same as that of SMR. However, it should be noted that in the case of AHMR, both positive and negative contributions come from a single layer of FM material. The former is uniform throughout the sample, whereas the latter is dependent on the distribution of reflected spin current from the edges/surfaces, which is determined by the relative orientation of the magnetization with respect to the sample geometry and current direction.

**Fig. 2:**
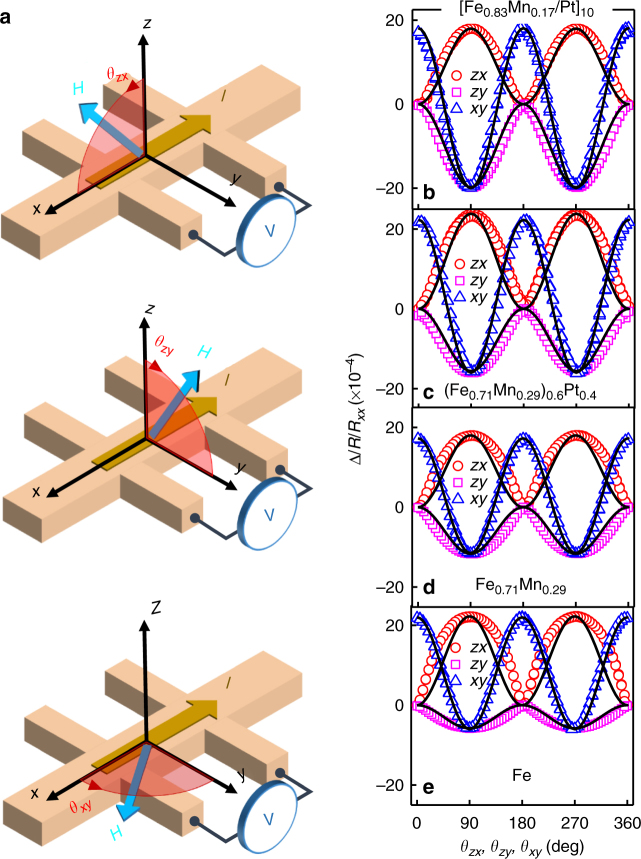
ADMR measurement geometry and typical results. **a** Schematic of measurement geometry. **b–d** ADMR measurement results: **b** [Fe_0.83_Mn_0.17_(0.6)Pt(0.4)]_10_/Pt(1) multilayer; **c** co-sputtered (Fe_0.71_Mn_0.29_)_0.6_Pt_0.4_(9); **d** Fe_0.71_Mn_0.29_(9); and **e** Fe. Solid lines in **b**–**e** are fittings based on the angle dependence

To experimentally characterize the AHMR, we fabricated four types of samples (see Fig. 1d for illustration of different types of sample structures). Al the samples were deposited on SiO_2_/Si substrates using sputtering and patterned into Hall bars by photolithography and liftoff techniques (see Methods for details). Through combined characterization of X-ray diffraction (XRD) and high-resolution scanning transmission electron microscopy, the samples were found to be polycrystalline with texture (see Supplementary Note 1). To minimize SHE from HM, all the samples were uncapped except for the Fe_1−*x*_Mn_*x*_/Pt multilayer samples, which ends automatically with a Pt layer. In order to prevent the sample from oxidation, this final Pt layer was intentionally made slightly thicker than the rest of the Pt layers inside the stack. In general, samples with *x* < 0.4–0.5 (depending on the structure) exhibit global FM behavior with an in-plane anisotropy at room temperature, and their temperature dependence of magnetization can be fitted well using a semi-empirical model^40^ (see Supplementary Note 1). Angle-dependent magnetoresistance (ADMR) measurements were performed by subjecting the sample to a rotational field of 30 kOe in the *zx*, *zy*, and *xy* planes, and measuring the longitudinal resistance under a DC current (see illustrations in Fig. 2a). Shown in Fig. 2b–e are the typical ADMR results for each type of sample, i.e., [Fe_0.83_Mn_0.17_(0.6)Pt(0.4)]_10_/Pt(1) multilayer, co-sputtered (Fe_0.71_Mn_0.29_)_0.6_Pt_0.4_(9), Fe_0.71_Mn_0.29_(9) and Fe(9) (the number inside the parentheses indicates thickness in nanometer, and the repeating period of the multilayer sample is 10). The field-dependent magnetoresistance (FDMR) results can be found in Supplementary Note 2. The angle *θ*_*ij*_ (*i, j* = *x*, *y*, *z*) denotes the angle between the rotating field and *i-*axis when the field rotates from *i-axis* to *j*-axis in the *ij* plane, e.g., *θ*_*xy*_ refers to the angle with respect to *x*-axis when the field rotates from *x*-axis to *y*-axis. As can be seen from these results, MR(*θ*_*zx*_) exhibits a sin^2^*θ*_*zx*_ symmetry, which is expected for conventional AMR in FM, whereas MR(*θ*_*zy*_) is similar to SMR in FM/HM bilayer with −sin^2^*θ*_*zy*_ symmetry (see solid lines in Fig. 2b–e for fitting). Furthermore, the magnitude of MR(*θ*_*xy*_) is the sum of the magnitude of MR(*θ*_*zx*_) and MR(*θ*_*zy*_) (the small difference may be due to slightly different saturation state in the three rotation directions). These features are in good agreement with the SMR observed in metallic FM/HM bilayers^41, 42^. However, what is striking is that the same type of MR behavior was observed in all four types of samples despite their significantly different sample structures. In fact, in the case of Fe_0.71_Mn_0.29_(9) and Fe(9), there is even no HM element involved at all. These results suggest that the MR(*θ*_*zy*_) observed in these samples must have an origin different from the SMR. We shall mention that similar MR(*θ*_*zy*_) was observed before in Fe/MgO^43^ and MgO/Fe/MgO or SiO_2_/Fe/SiO_2_^44^, but no unified explanation was given. Before ending this section, it is worth pointing out that there is a small deviation from the sin^2^*θzx* −sin^2^*θ*_*zy*_ dependence in the fitting data shown in Fig. 2b-e, and the deviation increases with the saturation magnetization. Numerical simulation by using the experimentally derived demagnetizing field in *z*-direction (*H*_d_) confirms that this is caused by the slight deviation of the magnetization direction from the external field direction when it is rotating in the *zx*-plane or *zy*-plane, though the latter (30 kOe in this case) is much higher than *H*_d_^45^. Nevertheless, this deviation only alters the shape of the ADMR curves, which does not affect the magnitude of the extracted MR ratio (see Supplementary Note 3).

### Correlation of MR(*θ*_*zy*_) and AHE

**Fig. 3:**
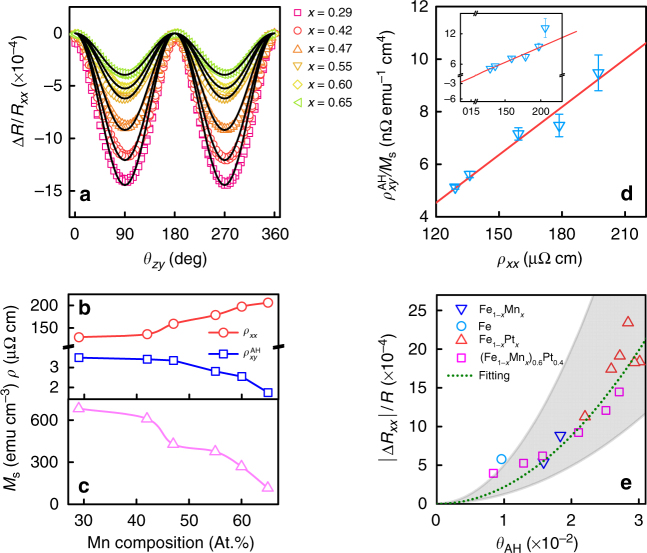
Correlation of MR(*θ*_*zy*_) and AHE. **a** MR(*θ*_*zy*_) results for co-sputtered (Fe_1−*x*_Mn_*x*_)_0.6_Pt_0.4_ with *x* = 0.22–0.65. **b**–**c** Mn composition dependence of *ρ*_*xx*_, $\rho _{xy}^{{\mathrm{AH}}}$ and *M*_s_, respectively. **d** $\rho _{xy}^{{\mathrm{AH}}}/M_{\mathrm{s}}$ as a function of *ρ*_*xx*_. **e** MR ratio as a function of *θ*_AH_ for a variety of samples including Fe, Fe_1−*x*_Pt_*x*_, (Fe_1-*x*_Mn_*x*_)_0.6_Pt_0.4_, and Fe_1−*x*_Mn_*x*_. Solid lines in **a** are fittings based on the angle dependence. Inset of **d** shows the full range of $\rho _{xy}^{{\mathrm{AH}}}/M_{\mathrm{s}}$ vs. *ρ*_*xx*_ plot, and solid line in **d** serves as a guide for the eye. The shadowed area in **e** is the calculated AHMR ratio range using different combinations of *β*: 0.4–0.6 and *l*_s_: 2–5 nm, and dotted line in **e** is the fitting results using *β* = 0.55 and *l*_s_ = 3.5 nm. The error bars in **d** are from the linear fitting to determine $\rho _{xy}^{{\mathrm{AH}}}$ from *ρ*_*xy*_

To examine whether the observed MR(*θ*_*zy*_) originates from AHE, we conducted scaling analysis by measuring MR(*θ*_*zy*_) in samples with fixed thickness but different AHE strength. Specifically, we varied the AHE strength by adjusting the Mn composition in FeMn/Pt, FeMnPt, and FeMn samples, and Pt composition in Fe_1−*x*_Pt_*x*_ samples (see Methods for more details). Although systematic studies have been performed on all these samples, here we only focus on the co-sputtered FeMnPt alloy samples in the main text as it is more representative as compared to the other three types of samples. The discussion on Fe_1−*x*_Mn_*x*_/Pt multilayer and Fe_1−*x*_Mn_*x*_ samples can be found in the Supplementary Note 4. The thickness of all samples is fixed at 9 nm. Figure 3a shows the ADMR when the magnetization rotates in the *zy*-plane, i.e., MR(*θ*_*zy*_), for co-sputtered (Fe_1−*x*_Mn_*x*_)_0.6_Pt_0.4_ samples with *x* = 0.22–0.65. As can be seen from the figure, the ADMR exhibits the same angle-dependence in the entire Mn composition range, though its magnitude decreases monotonically with increasing *x*. In addition, we investigated how longitudinal resistivity (*ρ*_*xx*_), anomalous Hall resistivity ($\rho _{xy}^{{\mathrm{AH}}}$) and *M*_s_ vary with *x* and the results are summarized in Fig. 3b, c, respectively. The $\rho _{xy}^{{\mathrm{AH}}}$ values in Fig. 3c are obtained from the raw Hall resistivity *ρ*_*xy*_ after subtracting out the contribution from ordinary Hall effect (see Supplementary Note 5). The anomalous Hall resistivity of FM is known to be proportional to the saturation magnetization in the same material system^31^, i.e., $\rho _{xy}^{{\mathrm{AH}}} = R_{\mathrm{s}}M_{\mathrm{s}}$, where *R*_s_ is the anomalous Hall coefficient and *M*_s_ is the saturation magnetization. It should be noted that among different material systems, such relation may not apply. The AHE in FM can originate from either intrinsic or extrinsic mechanisms. The former arises from the electronic band structure or Berry phase, whereas the latter is due to scattering of charges by impurity or defects with large spin–orbit coupling, via either skew-scattering or side-jump mechanism^31^. The extrinsic mechanism is presumably dominant in the present case considering the structure of the samples. In this case, it has been established previously that *R*_s _∝ *ρ*_*xx*_ for skew-scattering and $R_{\mathrm{s}} \propto \rho _{xx}^2$ for side-jump^31^, provided that *M*_s_ is constant. Since in the present case *M*_s_ is changing with Mn composition, in Fig. 3d, we plot $\rho _{xy}^{{\mathrm{AH}}}/M_{\mathrm{s}}$ as a function of *ρ*_*xx*_. As can be seen from the fitting, $\rho _{xy}^{{\mathrm{AH}}}/M_{\mathrm{s}}$ scales almost linearly with *ρ*_*xx*_ for *x* < 0.6, which suggests that skew scattering is indeed dominant in this case. The deviation for samples with *x* > 0.6 (see inset of Fig. 3d), is due to the weakening of FM order as evident from the drastic drop of *M*_s_ in Fig. 3c. In analogy to spin Hall angle (*θ*_SH_) of HM, we can define an anomalous Hall angle as $\theta _{{\mathrm{AH}}} = \frac{{\sigma _{xy}^{{\mathrm{AH}}}}}{{\sigma _{xx}}}$ or $\frac{{\rho _{xy}^{{\mathrm{AH}}}}}{{\rho _{xx}}}$, with $\sigma _{xy}^{{\mathrm{AH}}}$ and *σ*_*xx*_ the anomalous Hall and longitudinal conductivity of FM, respectively. By using the experimentally determined *θ*_AH_ values, in Fig. 3e, we plot the amplitude of MR(*θ*_*zy*_), i.e., Δ*R*/*R*_*xx*_, as a function of *θ*_AH_, for (Fe_1−*x*_Mn_*x*_)_0.6_Pt_0.4_ (squares). It is apparent that the curve is non-linear, suggesting that the observed ADMR is a second-order process of AHE, different from conventional AMR. It is worth noting that the maximum *θ*_AH_ value of 0.03 is comparable to the lower end of spin Hall angles reported for Pt (which itself is scattered over a large range)^46^, but is two times as large as *θ*_AH_ of NiFe/Pt bilayer^47^. In the case of NiFe/Pt, spin–orbit coupling at the interface has been cited as the cause for enhanced *θ*_AH_. In the present case, however, the enhancement of *θ*_AH_ in (Fe_1−*x*_Mn_*x*_)_0.6_Pt_0.4_ is presumably due to Pt atoms uniformly distributed in the alloy films. In addition to (Fe_1−*x*_Mn_*x*_)_0.6_Pt_0.4_, we also show the results for Fe_1−*x*_Mn_*x*_, Fe_1−*x*_Pt_*x*,_ and Fe in Fig. 3e (represented by different symbols). We will discuss these results shortly after presenting the analytical model. Besides these samples, some other common FM and antiferromagnetic materials including Co, NiFe, and Ir_0.2_Mn_0.8_ were also examined, but they all exhibit a *θ*_AH_ at least one order of magnitude smaller, and therefore either very small or different MR(*θ*_*zy*_) behavior was observed (see Supplementary Note 6). This is expected because the size of $\rho _{xy}^{{\mathrm{AH}}}$ in transition metals typically follows the order: Fe >> Co > Ni^48–50^. As discussed in the Supplementary Note 7, field misalignment is not able to account for the magnitude of the measured MR(*θ*_*zy*_) curves. Apart from the field misalignment, another possible source for the MR(*θ*_*zy*_) observed is the geometric size effect (GSE) related AMR. However, if this is indeed the case, one would expect a same temperature dependence of MR(*θ*_*zy*_) and MR(*θ*_*zx*_). But, as shown in Supplementary Fig. 12, we observed a different temperature dependence for MR(*θ*_*zy*_) and MR(*θ*_*zx*_) in the FeMnPt and Fe samples, but same temperature dependence in the Py control sample which has a much smaller AHE. In view of these results, both field misalignment and GSE-related AMR can be ruled out as the origin of the observed MR(*θ*_*zy*_).

### Derivation of AHMR

**Fig. 4:**
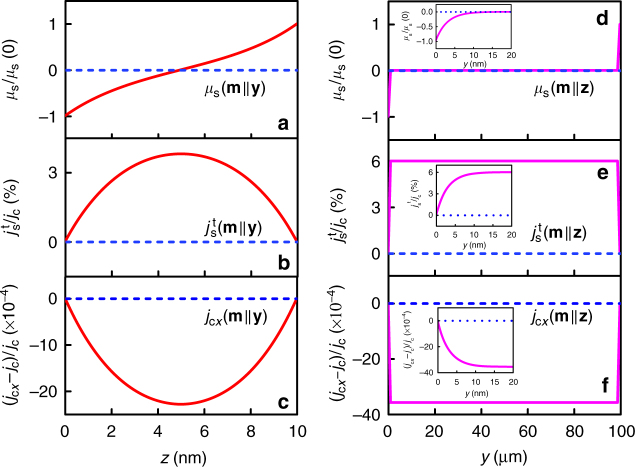
Modulation of the spin accumulation, transverse spin current, longitudinal charge current by AHE in FM. **a**–**c** normalized distributions along *z*-direction for the cases of **m**||**y**: **a** spin accumulation *μ*_s_/*μ*_s_(0); **b** spin current $j_{\mathrm{s}}^{\mathrm{t}}/j_{\mathrm{c}}$; and **c** charge current (*j*_c*x*_−*j*_c_)/*j*_c_. **d**–**f** normalized distributions along *y*-direction for the cases of **m**||**z**: **d** spin accumulation *μ*_s_/*μ*_s_(0); **e** spin current $j_{\mathrm{s}}^{\mathrm{t}}/j_{\mathrm{c}}$; and **f** charge current (*j*_c*x*_−*j*_c_)/*j*_c_. Dashed lines in **a**–**f** are the reference when no AHE is present in the sample. Insets of **d**–**f** are the distributions near the vicinity of the edges

In order to have a quantitative understanding of the results shown in Fig. 3e, we derive the analytical equation for MR in a single FM layer by including the AHE and its inverse effect (see Supplementary Note 8). As discussed, a transverse spin current $j_{\mathrm{s}}^{\mathrm{t}}$ is generated in the direction of **m** × **j**_**c**_ when the charge current **j**_**c**_ flows in an FM, where **m** is the magnetization direction. In the case of bulk, $j_{\mathrm{s}}^{\mathrm{t}}$ is uniformly distributed inside the sample, which gives an extra resistance due to the additional opposite charge current induced by the inverse AHE. However, due to spin accumulation and backflow of spin current from the boundary, the situation changes when the sample has a finite dimension in the $j_{\mathrm{s}}^{\mathrm{t}}$ flowing direction. As derived in Supplementary Note 8, this will lead to a MR that is given in the general form of

1 $$\rho _{xx} = \rho _0\left( {1 + Am_x^2 + (\theta _{{\mathrm{AH}}}/\beta )^2\left[ {m_z^2 + \left( {1 - \frac{{2l_{\mathrm{s}}}}{d}{\mathrm{tanh}}(\frac{d}{{2l_{\mathrm{s}}}})} \right)m_y^2} \right]} \right)$$

where *d* and *l*_s_ are the thickness and spin diffusion length of FM, respectively. *β* is the polarization for longitudinal conductivity, *θ*_AH_ is the anomalous Hall angle, and *A* is the AMR ratio. Apparently, Eq. (1) contains both AMR and AHMR contributions. In order to have an anatomical view of the spin–charge conversion process that leads to the AHMR, we plot the normalized spin accumulation *μ*_s_/*μ*_s_(0), spin current $j_{\mathrm{s}}^{\mathrm{t}}/j_{\mathrm{c}}$, and charge current (*j*_c*x*_−*j*_c_)/*j*_c_ in Fig. 4a, c for **m**||**y**, and in Fig. 4d–f for **m**||**z**, respectively. Insets of Fig. 4d–f are the distributions near the sample edges. For the case of **m**||**y**, $j_{\mathrm{s}}^{\mathrm{t}}$ flows in *z*-direction with the spin polarization in *y*-direction, and it accumulates at the top and bottom surfaces. Under the boundary condition $j_{\mathrm{s}}^{\mathrm{t}}$ = 0 at both surfaces, the spatial distribution of spin accumulation (*μ*_s_), transverse spin current ($j_{\mathrm{s}}^{\mathrm{t}}$), and longitudinal charge current (*j*_c*x*_) in *z*-direction are given by

2 $$\mu _{\mathrm{s}}\left( z \right) = \frac{{2el_{\mathrm{s}}j_{\mathrm{c}}}}{{\sigma _{xx}}}\frac{{\theta _{{\mathrm{AH}}}}}{\beta }\frac{{\cosh \left( {\frac{z}{{l_{\mathrm{s}}}}} \right) - \cosh \left( {\frac{{z - d}}{{l_{\mathrm{s}}}}} \right)}}{{{\mathrm{sinh}}(d/l_{\mathrm{s}})}}$$

3 $$j_{\mathrm{s}}^{\mathrm{t}}\left( z \right) = \frac{{j_{\mathrm{c}}\theta _{{\mathrm{AH}}}}}{\beta }\left[ {1 - \frac{{\sinh \left( {\frac{z}{{l_{\mathrm{s}}}}} \right) - {\mathrm{sinh}}\left( {\frac{{z - d}}{{l_{\mathrm{s}}}}} \right)}}{{{\mathrm{sinh}}(d/l_{\mathrm{s}})}}} \right]$$

4 $$j_{{\mathrm{c}}x}\left( z \right) = j_{\mathrm{c}} - j_{\mathrm{c}}\left( {\frac{{\theta _{{\mathrm{AH}}}}}{\beta }} \right)^2\left[ {1 - \frac{{\sinh \left( {\frac{z}{{l_{\mathrm{s}}}}} \right) - {\mathrm{sinh}}\left( {\frac{{z - d}}{{l_{\mathrm{s}}}}} \right)}}{{{\mathrm{sinh}}(d/l_{\mathrm{s}})}}} \right]$$

where *j*_c_ is the original applied charge current in *x*-direction, *σ*_*xx*_ is the conductivity, *e* is the electron charge, and the rest of parameters are already defined as above. The second term in the brackets of Eqs. (3) and (4) is resulted from the backflow of spin current induced by the spin accumulation described by Eq. (1). The same set of equations applies to the case when **m**||**z** except that *d* is replaced by the sample width (*w*), and the spatial distribution is along *y*-direction. In the calculations, we have used *d* = 10 nm, *w* = 100 μm, *β* = 0.5, *l*_s_ = 3 nm, and *θ*_AH_ = 0.03. The values used for polarization and spin diffusion length are within the range of those reported in FMs^51^. As shown in Fig. 4b for **m**||**y**, where the dashed lines are added as a reference to show the case when AHE is absent in the sample, due to the comparable scale of *d* and *l*_s_, the backflow spin current cancels $j_{\mathrm{s}}^{\mathrm{t}}$ largely throughout the sample (see Fig. 1a for illustration). In contrast, in the case of **m**||**z**, the cancellation is mainly confined in the vicinity of the two side edges: $j_{\mathrm{s}}^{\mathrm{t}}$ in the remaining region remains almost intact because *w* >> *l*_s_ (see Fig. 4e and Fig. 1b). It is this difference in the cancellation of $j_{\mathrm{s}}^{\mathrm{t}}$ that leads to the different degree of charge current correction, which consequently results in the different resistance for **m**||**y** and **m**||**z**: the origin of AHMR. On the other hand, the AHE is absent when **m**||**x**, and therefore no transverse spin current/spin accumulation nor redistribution of charge current occurs in this case. Although the AHE does not come into play when **m**||**x**, the conventional AMR still exits and gives rise to an increase in resistance, which is revealed by the second term in Eq. (1). Therefore, the AHMR, which is given by the third term of Eq. (1), has the same angular dependence as SMR, in qualitative agreement with the experimental data shown in Figs. 2b–d and 3a. Notably, the size of AHMR ratio, given by $\left( {\frac{{\theta _{{\mathrm{AH}}}}}{\beta }} \right)^2\frac{{2l_{\mathrm{s}}}}{d}{\mathrm{tanh}}\left( {\frac{d}{{2l_{\mathrm{s}}}}} \right)$, exhibits a quadratic relationship with *θ*_AH_, which is in good agreement with the ADMR data shown in Fig. 3e. These results affirm our argument that the ADMR in *zy*-plane is caused by the AHE and its inverse in the FM layer, i.e., the AHMR. It is apparent from Eq. (1) that, in addition to the experimentally determined anomalous Hall angle *θ*_AH_, the magnitude of AHMR is also directly dependent on *β* and *l*_s_, which are not available experimentally for most of the materials under investigation except for Fe. And in fact even for Fe, the reported values are scattered in a large range depending on the techniques used, film thickness, techniques used to prepare the film, etc. Therefore, in Fig. 3e, we first plot the range of AHMR calculated by using *β* = 0.4−0.6 and *l*_s_ = 2−5 nm (shadowed region). The lower and upper boundary denoted the minimum and maximum values obtained by *β* = 0.6, *l*_s_ = 2 nm and *β* = 0.4, *l*_s_ = 5 nm, respectively. The range for *β* and *l*_s_ are chosen to cover most of the ferromagnets. Therefore, besides (Fe_1−*x*_Mn_*x*_)_0.6_Pt_0.4_, we also added the results for Fe_1−*x*_Mn_*x*_, Fe_1−*x*_Pt_*x*_, and Fe in the same figure (all have a thickness of 9 nm). The anomalous Hall angle were varied by changing either the Mn or Pt composition (except for Fe). Despite the variation in composition, the AHMR for all these Fe-based films indeed show a quadratic dependence on *θ*_AH_, as manifested in the dotted line, which is the fitting result for (Fe_1−*x*_Mn_*x*_)_0.6_Pt_0.4_ obtained by using *β* = 0.55 and *l*_s_ = 3.5 nm. As we will discuss shortly, similar range of values can also fit the thickness dependence of AHMR as predicted by Eq. (1).

### Thickness dependence of AHMR

**Fig. 5:**
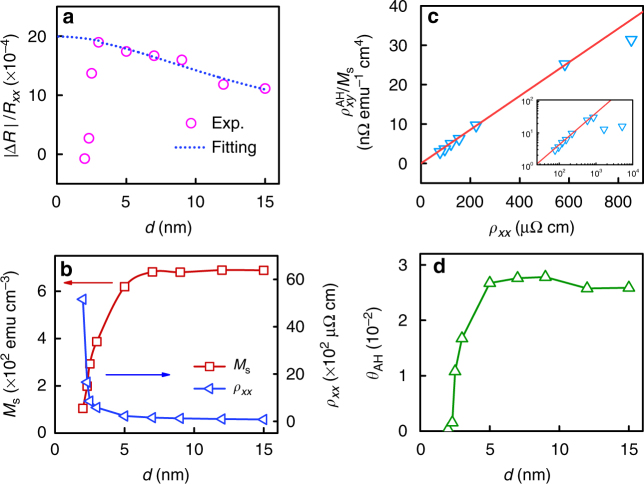
Thickness dependence of AHMR and AHE in co-sputtered (Fe_0.71_Mn_0.29_)_0.6_Pt_0.4_ samples. **a** AHMR ratio for (Fe_0.71_Mn_0.29_)_0.6_Pt_0.4_ with *d* = 2–15 nm, and the fitting using Eq. (4) with fixed *θ*_AH_ for *d* = 5–15 nm (dotted line). **b** Thickness dependence of *M*_s_ and *ρ*_*xx*_. **c** $\rho _{xy}^{{\mathrm{AH}}}/M_{\mathrm{s}}$ as a function of *ρ*_*xx*_ in the linear range. **d** Thickness dependence of *θ*_AH_. Inset of **c** shows the full range of $\rho _{xy}^{{\mathrm{AH}}}/M_{\mathrm{s}}$ vs. *ρ*_*xx*_ plot, and solid line in **c** serves as a guide for the eye

Figure 5a shows the experimentally observed thickness dependence of AHMR for (Fe_0.71_Mn_0.29_)_0.6_Pt_0.4_ with *d* = 2–15 nm. Instead of a monotonic decrease of AHMR with increasing *d* as predicted by the theoretical model, the experimental value increases sharply at small thickness, peaks at around *d* = 3 nm, and then decreases slowly as *d* increases. There are two possible reasons that cause the deviation from theoretical model at small thickness: one is the sharp increase of resistivity due to surface scattering and the other is the decrease of magnetization due to finite size effect. When the thickness of a thin film becomes smaller than or comparable to the electron mean free path, its resistivity scales with the thickness as $\rho _{xx} = \rho _{xx0}\left[ {1 + \left( {1 - p} \right)\frac{{3l_{\mathrm{f}}}}{{8\left( {d - d_0} \right)}}} \right]$, here *ρ*_*xx*0_ is the bulk resistivity, *p* is the specular reflectivity, *d*_0_ is the roughness, and *l*_f_ is the electron mean free path^52^. For surface with finite roughness or small *p*, the resistivity will increase sharply when *d* < *l*_f_ or *d*_0_. Figure 5b shows *ρ*_*xx*_ and *M*_s_ as a function of *d*. As expected, *ρ*_*xx*_ increases, whereas *M*_s_ decreases sharply at small thickness. It is interesting to note that *M*_s_ starts to decrease at a larger thickness than *ρ*_*xx*_, understandably from the difference in length scale that governs the resistivity and magnetization of thin films. More discussion on the effect of thin film roughness can be found in Supplementary Note 9. In Fig. 3, we found that the relation $\theta _{xy}^{{\mathrm{AH}}} \propto M_{\mathrm{s}}$ holds for most of the Mn composition range for FeMnPt, suggesting that the AHE is dominated by skew scattering^31^. For comparison, we plot, in Fig. 5c, $\rho _{xy}^{{\mathrm{AH}}}/M_{\mathrm{s}}$ as a function of *ρ*_*xx*_ for samples with different thicknesses. A nearly perfect linear relation is obtained when *d* > 3 nm. However, at *d* < 3 nm (see inset of Fig. 5c), a sublinear relation appears, suggesting gradual weakening of AHE in this region. This is understandable because, in this region, surface scattering dominates the electrical transport; but compared to bulk scattering, surface scattering may not be an efficient mechanism for AHE since it is mostly spin-independent. Surface effect was not taken into account when deriving Eq. (1); therefore, strictly speaking, it does not apply to the case when the film thickness becomes comparable to or smaller than the spin diffusion length, which is usually larger than the mean-free path. Figure 5d shows the experimentally determined anomalous Hall angle *θ*_AH_ as a function of *d*. It is almost a constant above *d* = 5 nm, but decreases rapidly below this thickness. If we take the average value of *θ*_AH_ = 0.026 for *d* = 5–15 nm, we are able to fit the MR-dependence on thickness well using Eq. (1) for *d* > 5 nm. The fitting curve is shown in Fig. 5a as dotted line, in which we have used *β* = 0.58, *l*_s_ = 4.5 nm. These values are in the same range as those that are used for the fitting in Fig. 3 (dotted line) though they are not exactly the same (presumably due to thickness-dependent surface effect). The deviation at very small thickness corresponds to the region where surface scattering becomes dominant, leading to a sharp increase of resistivity and decrease of *β*^53^. In the same region, *M*_s_ decreases rapidly as well due to the decrease in Curie temperature. As the decrease of both *M*_s_ and *β* leads to a more rapid decrease of *θ*_AH_ as compared to *β* itself, the AHMR diminishes rapidly at small thickness. Therefore, in addition to the quadratic dependence on *θ*_AH_ presented in Fig. 3e, the thickness dependence of AHMR shown in Fig. 5a also strongly supports the AHE origin of MR(*θ*_*zy*_). To further substantiate this argument, we have fabricated another series of (Fe_0.71_Mn_0.29_)_0.6_Pt_0.4_ sample, as well as a series of Fe samples in the thickness range of 5–20 nm. Again, a monotonically decreasing MR(*θ*_*zy*_) ratio is obtained in the entire thickness range of *d* = 5–20 nm for both series of samples (see Supplementary Note 10 for more details). All these results combined confirm the reproducibility of the experimental results and validity of the AHMR scenario presented in this work.

## Discussion

As discussed above, the AHMR has been observed in co-sputtered (Fe_1−*x*_Mn_*x*_)_0.6_Pt_0.4_, Fe_1−*x*_Mn_*x*_/Pt multilayer, Fe_1−*x*_Mn_*x*_ with *x* = 0.17–0.65 and Fe. The resistivity and magnetization of these samples were varied systematically using both the Mn composition and layer thickness, which in turn allows us to use scaling analysis to examine the AHE origin of the observed MR. Both the magnitude and thickness dependence of the MR (>spin diffusion length) are in reasonable agreement with those calculated from an analytical model based on the drift-diffusion formalism. The FeMnPt-based materials were chosen because they exhibit a relatively large AHE, and importantly the size of AHE can be tuned by adjusting the chemical composition. However, on the other hand, these materials are relatively new and their magnetic and electrical properties are less understood across the composition range. It will be of importance to confirm if the AHMR is also present in others whose magnetic properties have been thoroughly investigated and well understood. One possible approach is to tune the AHE of Py by adjusting the Fe composition, and see if there is any correlation between AHE and MR(*θ*_*zy*_), though the weak AHE may pose a challenge in interpreting the experimental data. Another candidate for investigating the AHMR is the class of materials with giant AHE reported recently^54, 55^. In addition to electrical measurement, it would also be of interest to probe the AHE-generated spin current directly using magneto-optical technique and correlate it with the MR data. We believe that the results described in this work demonstrate the importance of AHE as an alternate tool for studying spin–charge interconversion in magnetic materials and its potential in spintronic applications.

## Methods

### Sample preparation

All samples were deposited on SiO_2_(300 nm)/Si substrates using DC magnetron sputtering with a base and working pressure of 2 × 10^−8^ and 3 × 10^−3^ Torr, respectively. Fe_1−*x*_Mn_*x*_ (or Fe_1−*x*_Pt_*x*_) films were prepared by co-sputtering of Fe_0.8_Mn_0.2_ and Mn targets (or Fe and Pt targets). [Fe_1−*x*_Mn_*x*_(0.6)/Pt(0.4)]_10_ multilayer samples were prepared by sequential deposition of Fe_1−*x*_Mn_*x*_ and Pt layers in a repeated manner, while (Fe_1−*x*_Mn_*x*_)_0.6_Pt_0.4_ samples were deposited by co-sputtering of Fe_0.8_Mn_0.2_, Mn, and Pt. The chemical composition of all the samples was determined by X-ray photoelectron spectroscopy (XPS). Standard photolithography and liftoff techniques were used to fabricate the Hall bar, which consists of a mesa 1.1 mm long and 100 μm wide, with four 50 μm wide and 200 μm long protrusions placed at sides of the bar as voltage probes. A Microtech laserwriter system with a 405 nm laser was employed to directly expose the substrates after coating the positive photoresist Microposit S1805. After exposure, the substrates were then soaked in developer MF319 to form the Hall bar pattern. After deposition using sputtering, the photoresist was removed by the mixture of PG remover and acetone, and the metallic patterns are left on the substrates.

### Characterization

Structural properties of the samples were characterized using a Rigaku XRD system with Cu Kα radiation. XPS was performed on PHI Quantera II XPS Scanning Microprobe from Ulvac-PHI with a beam spot size of 50 μm. In addition, high-resolution scanning transmission electron microscopy (STEM, a JEOL ARM200F) was employed to directly image the multilayer samples. Magnetic properties were characterized using a quantum design vibrating sample magnetometer (VSM) with the samples cut into a size of 4 mm × 3 mm. The resolution of the system is better than 6 × 10^−7^ emu. The electrical measurements were also performed using the same Quantum Design system at a bias current of 100 μA.

### Data availability

The data that support the findings of this study are available from the corresponding author on request.

## Electronic supplementary material

Supplementary Information
