# Supplementary material for: Anomalous Hall magnetoresistance in a ferromagnet
Source: Nat Commun. 2018 Jun 8;9:2255. doi: 10.1038/s41467-018-04712-9 (PMC5993777; doi:10.1038/s41467-018-04712-9)
Supplement: Supplementary file 1 — Supplementary Information [file 41467_2018_4712_MOESM1_ESM.pdf]

Supplementary Information for “Anomalous Hall magnetoresistance in a ferromagnet”

Yumeng Yang et al.

Supplementary Note 1. Structural and magnetic properties of coupon films

Supplementary Figure 1a shows the X-ray diffraction (XRD) patterns of four coupon films selected from the three types of FMs: $[Fe_{0.83}Mn_{0.17}(0.6)/Pt(0.4)]_{20}$ multilayer, $(Fe_{0.71}Mn_{0.29})_{0.6}Pt_{0.4}(20)$, $Fe_{0.71}Mn_{0.29}(20)$ and $Fe_{0.4}Mn_{0.6}(20)$, covering the peak range of fcc Pt (111) at 39.8°, fcc γ-$Fe_{1-x}Mn_x$ (111) at 43.5°, and bcc α-$Fe_{1-x}Mn_x$ (110) at 44.7°. To obtain moderate X-ray counts, the thickness of the films were kept at 20 nm, which is thicker than the patterned samples used for electrical measurements. It has been reported that $Fe_{1-x}Mn_x$ with $0.3 < x < 0.7$ is stable in γ-phase, while for $x < 0.3$ it undergoes a transition to α-phase with the increase of Fe composition[1, 2]. The diffraction peak of $Fe_{0.71}Mn_{0.29}$ appears at 44.7°, suggesting that the film is dominantly in bcc α-phase (110), while the shift of the peak to 43.2° for $Fe_{0.4}Mn_{0.6}$ agrees with these previous reports that fcc γ-phase (111) becomes dominant when x is below 0.3. On the other hand, the peaks at 40.9° - 41.1° observed in the multilayer and co-sputtered samples correspond to neither fcc Pt (111) at 39.8° nor $Fe_{1-x}Mn_x$ peaks. In the case of co-sputtered sample, it is understandable because Pt and $Fe_{1-x}Mn_x$ mixes uniformly to form an alloy. It is interesting to note that the diffraction peak for the multilayer appears at almost the same position as that of the co-sputtered film.

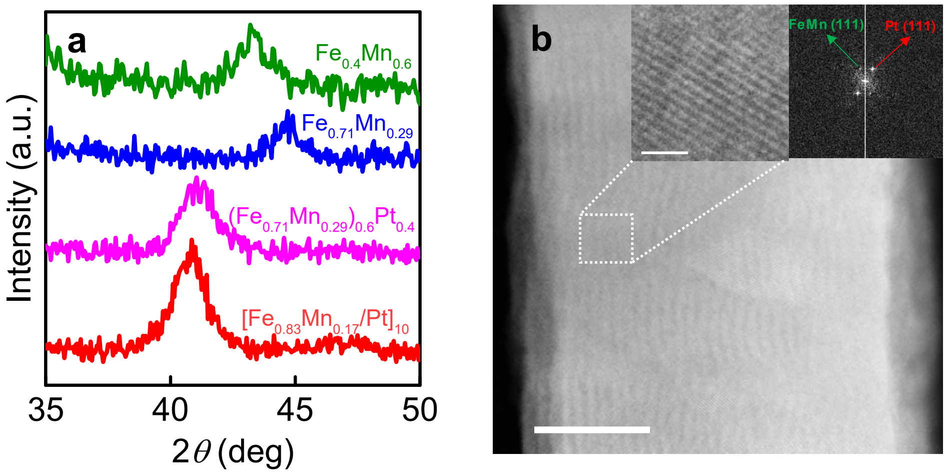

Supplementary Figure 1. Structural characterizations of coupon films. **a**, XRD pattern for $[Fe_{0.83}Mn_{0.17}(0.6)/Pt(0.4)]_{20}$, $(Fe_{0.71}Mn_{0.29})_{0.6}Pt_{0.4}(20)$, $Fe_{0.71}Mn_{0.29}(20)$, and $Fe_{0.4}Mn_{0.6}(20)$. **b,** STEM HAADF image of cross section of $[Fe_{0.5}Mn_{0.5}(0.6)/Pt(0.6)]_{30}$. Inset in **b** is an enlargement of the area enclosed by the dashed square and the FFT pattern of the same area. The white scale bar in **b** is 10 nm, and that in the inset is 1 nm.

To confirm the structure of the multilayer sample, we employed high resolution scanning transmission electron microscopy (STEM, a JEOL ARM200F) to directly image a multilayer sample consisting of $[Fe_{0.5}Mn_{0.5}(0.6)/Pt(0.6)]_{30}$. For a better recognition of the individual layer, the Pt thickness is slightly increased to be equal to that of FeMn, and the repetition period is also increased to 30. Supplementary Figure 1b is the cross-section high-angle annular dark field (HAADF) image of the sample, which shows a clear layer-by-layer structure except for some waviness of the layers. The HAADF image shows strong Z-contrast (Z being atomic number) and the Pt layers image bright. The waviness may come from the roughness of the SiO_2/Si substrate since no seed layer was deposited and the thickness of the individual layers is in the sub-nm range. As a result, it is difficult to separate and identify the individual layers clearly in the image. Fast Fourier transformation (FFT) was performed on the selected area enclosed by the dashed square. As shown in the inset of Supplementary Figure 1b, the FFT pattern has sharp spots, which correspond to fcc γ-$Fe_{0.5}Mn_{0.5}$ (111) and fcc Pt (111), respectively. This agrees with the above analysis based on XRD. The STEM and XRD results suggest that all the three types of samples are textured polycrystalline films, though the multilayer sample exhibits periodic structure with some waviness.

Supplementary Figures 2a – 2c show the in-plane *M-H* loops for the three types of samples at room temperature: $[Fe_{1-x}Mn_x(0.6)/Pt(0.4)]_{10}/Pt(1)$, $(Fe_{1-x}Mn_x)_{0.6}Pt_{0.4}(9)$ and $Fe_{1-x}Mn_x(9)$ with $x = 0.17 – 0.65$. All the samples exhibit in-plane magnetic anisotropy as evident from the nearly square-like *M-H* loops and small coercivity. The FM order gradually weakens with the increase of Mn composition, as reflected in the decrease of saturation magnetization M_s. As mentioned above, $Fe_{1-x}Mn_x$ undergoes a transition from bcc α-phase to fcc γ-phase with the increase of Mn composition. The former is known to be an FM phase whereas the latter is an antiferromagnet (AF)[1, 2]. Therefore, the decrease of M_s can be understood as the gradual increase of AF ordering against the FM region as Mn composition increases. The inclusion of Pt in both the multilayer and co-sputtered samples extends the AF/FM boundary towards higher Mn

composition due to proximity effect between Pt and $Fe_{1-x}Mn_x$[3-5]. This is the reason why some of the co-sputtered and multilayer films still exhibit ferromagnetic properties in the range $x = 0.5 – 0.6$.

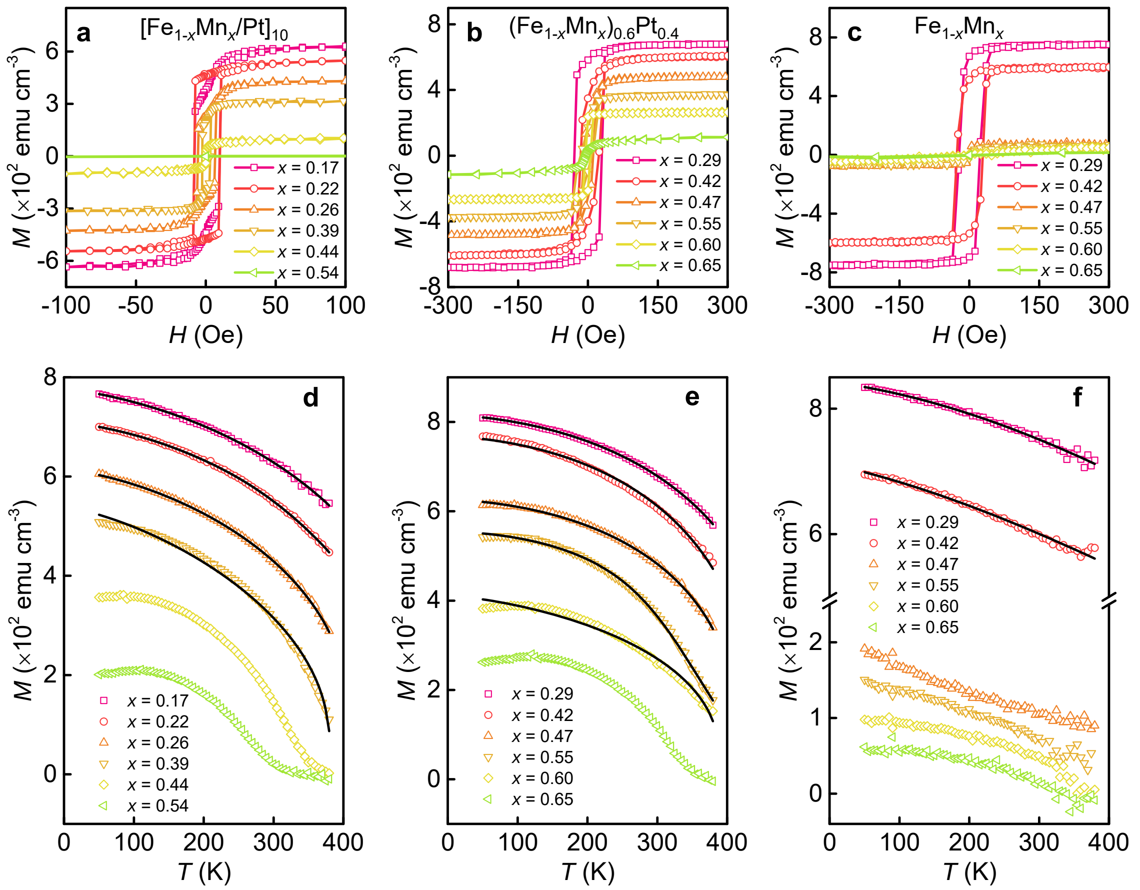

Supplementary Figure 2. Magnetic properties of coupon films. a - c, *M-H* loops: **a,** $[Fe_{1-x}Mn_x(0.6)/Pt(0.4)]_{10}/Pt(1)$; **b,** $(Fe_{1-x}Mn_x)_{0.6}Pt_{0.4}(9)$; **c,** $Fe_{1-x}Mn_x(9)$. **d - f,** *M-T* curves: **d,** $[Fe_{1-x}Mn_x(0.6)/Pt(0.4)]_{10}/Pt(1)$; **e,** $(Fe_{1-x}Mn_x)_{0.6}Pt_{0.4}(9)$; **f,** $Fe_{1-x}Mn_x(9)$. Solid lines in **d - f** are fittings based on Supplementary Equation 1.

To gain more insight into the magnetic properties, we examined the temperature dependence of magnetization in these samples, and the results are summarized in Supplementary Figures 2d – 2f. As with most ferromagnetic materials, the M_s decreases with the increase of temperature. It is also apparent that the Curie temperature (T_C) decrease with increasing Mn composition. For a more quantitative understanding, we invoke the semi-empirical model developed by Kuz'min[6, 7], which turned out to be very successful in fitting the *M-T* curves of many different types of magnetic materials, to fit these curves

in Supplementary Figures 2d – 2f. According to this model, the temperature dependent magnetization of FM is given by

$$M(T) = M(0)\left[1 - s\left(\frac{T}{T_C}\right)^{3/2} - (1-s)\left(\frac{T}{T_C}\right)^{5/2}\right]^b \qquad (1)$$

where $M(0)$ is the magnetization at $T = 0$ K, T_C is the Curie temperature, s is the so-called shape parameter with a value in the range of 0 - 2.5, and b is the critical exponent whose value is determined by the universality class of the material: 0.125 for two-dimensional Ising, 0.325 for three-dimensional (3D) Ising, 0.346 for 3D XY, 0.365 for 3D Heisenberg, and 0.5 for mean-field theory[8]. On the other hand, for surface magnetism, b is in the range of 0.75–0.89[9, 10]. Considering the 3D ferromagnetic nature of these samples, we fixed b at 0.365 and fitted the *M-T* curves by optimizing the remaining parameters. The values are summarized in Supplementary Table 1. The fitting is generally good for samples with low Mn compositions (around $x = 0.4$ for FeMn and FeMn/Pt multilayers and $x = 0.6$ for FeMnPt alloy). However, at higher Mn compositions, the *M-T* curves tend to deviate from that described by Supplementary Equation 1 with the s value out of its normal range; therefore we did not fit these curves and leave the parameters as N.A. in the table. The deviation is presumably caused by the onset of AFM ordering, and therefore, understandably, their *M-T* curves will not follow that of the FM. According to M. D. Kuz`min *et al.*, for 3D Heisenberg magnets, s is determined by the dependence of exchange interaction on interatomic distance[7]. It is generally positive with a small s (< 0.4) corresponding to metallic FMs with long-range ferromagnetic ordering and high T_C, whereas a large s (> 0.8) is indicative of competing exchange interactions and the resultant material typically has a low T_C. Following this reasoning, among the three types of samples, the co-sputtered samples behave more like a metallic FM than the other two types of samples do, and therefore, in the main text, the analysis on AHE and MR data have been focused on the co-sputtered samples.

Supplementary Table 1. Summary of the fitting parameters using Supplementary Equation 1 for the three types of samples. N.A. is the abbreviation for not available.

Type	x	$M(0)$ (emu cm^{-3})	s	T_C (K)
$Fe_{1-x}Mn_x$/Pt	0.17	774	0.96	516
	0.22	709	0.93	468
	0.26	613	1.07	417
	0.39	536	1.48	382
	0.44	N.A.	N.A.	N.A.
	0.54	N.A.	N.A.	N.A.
$(Fe_{1-x}Mn_x)_{0.6}Pt_{0.4}$	0.29	815	0.47	481
	0.42	767	0.45	441
	0.47	626	0.45	419
	0.55	576	1.10	386
	0.60	410	1.07	392
	0.65	N.A.	N.A.	N.A.
$Fe_{1-x}Mn_x$	0.29	840	1.38	844
	0.42	703	1.20	650
	0.47	N.A.	N.A.	N.A.
	0.55	N.A.	N.A.	N.A.
	0.60	N.A.	N.A.	N.A.
	0.65	N.A.	N.A.	N.A.

Supplementary Note 2. Field dependent magnetoresistance (FDMR) measurement results

As a supplementary reference, FDMR results are shown in Supplementary Figures 3a – 3l for the $[Fe_{0.83}Mn_{0.17}(0.6)Pt(0.4)]_{10}$/Pt(1) multilayer, co-sputtered $(Fe_{0.71}Mn_{0.29})_{0.6}Pt_{0.4}(9)$, $Fe_{0.71}Mn_{0.29}(9)$ and Fe(9) with applied sweeping field in x-, y- and z-axis, respectively. The data are thus denoted as H_x, H_y, and H_z FDMR curves, respectively. As can be seen from Supplementary Figures 3a, 3d, 3g and 3j, the shape of

the H_x and H_y FDMR curves resembles that of the conventional AMR, which gives $\rho_x > \rho_y$ with ρ_x (ρ_y) the resistivity when $\mathbf{m} \parallel \mathbf{x}$ ($\mathbf{m} \parallel \mathbf{y}$). For the H_z FDMR curves, the negative MR above saturation field

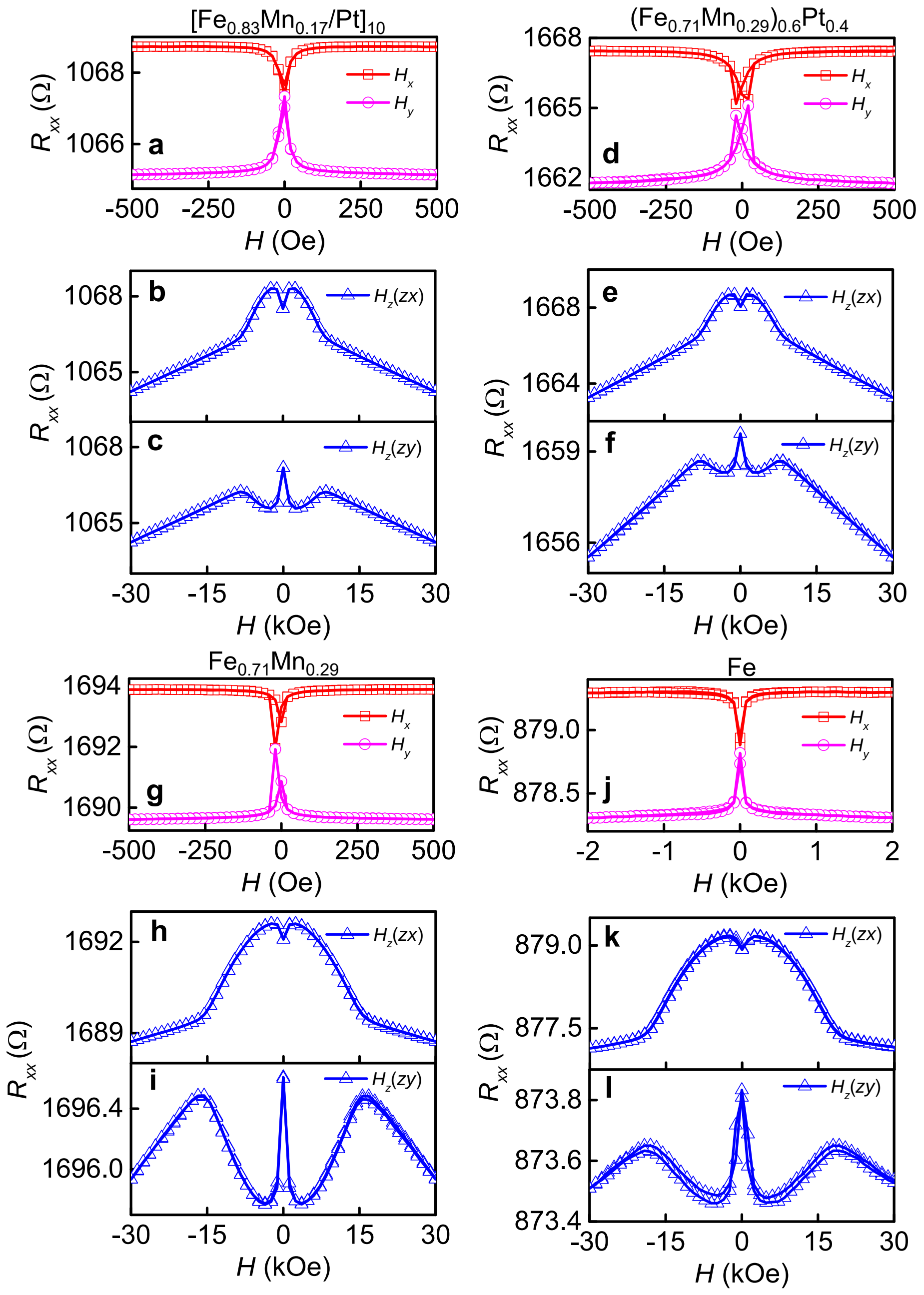

Supplementary Figure 3. FDMR curves for different types of samples. a - c, [$Fe_{0.83}Mn_{0.17}$(0.6)/Pt(0.4)]$_{10}$/Pt(1); **d - f,** $(Fe_{0.71}Mn_{0.29})_{0.6}Pt_{0.4}$(9); **g - i,** $Fe_{0.71}Mn_{0.29}$(9); **j - l,** Fe(9). The legends H_x, H_y and H_z denotes the FDMR curves obtained when the field is swept in *x*, *y*, and *z*-axis direction, respectively; and *zx* (or *zy*) in the parenthesis after H_z indicates the misalignment of H_z from *z*-axis towards *x*-axis (or *y*-axis).

(around 15 kOe) is attributed to the so called spin disorder MR[11]. Below saturation field, depending on the H_z field misalignment direction, two different shapes can be obtained. When H_z is misaligned from z-axis towards x-axis, the additional x-component of field helps rotate the magnetization in zx plane, and an M shaped MR curve is observed (Supplementary Figures 3b, 3e, 3h, 3k). On the other hand, when it is misaligned from z-axis towards y-axis, the magnetization is rotated in zy plane by the additional y-component, and a W shaped MR curve is observed (Supplementary Figures 3c, 3f, 3i, 3l). In view of these shapes and the magnetization positions, it can be inferred that $\rho_x > \rho_z > \rho_y$ with ρ_z the resistivity when $\mathbf{m} \parallel \mathbf{z}$. This relation is in agreement with the above H_x and H_y FDMR curves and ADMR results in Fig. 2 of the main text. To support the explanation, we performed macro-spin simulation for the H_z case following the approach described in our previous work[3]. Taking into consideration the misalignment, the applied field $\mathbf{H}$ is expressed as:

$$\mathbf{H} = \left(H\sin\delta\cos\chi, H\sin\delta\sin\chi, H\cos\delta\right) \tag{2}$$

here δ and χ are the misalignment polar and azimuth angles, respectively. The free energy density (normalized to saturation magnetization) is given by

$$E = \frac{H_\mathrm{d}}{2}\cos^2\theta - H(\sin\theta\cos\varphi\sin\delta\cos\chi + \sin\theta\sin\varphi\sin\delta\sin\chi + \cos\theta\cos\delta) - \frac{H_k}{2}\sin^2\theta\cos^2\varphi \tag{3}$$

where θ and φ are the polar and azimuth angles of the magnetization, respectively; $H_k = 2K_\mathrm{u}/M_\mathrm{s}$ is the anisotropy field; and H_d is the demagnetizing field. Here, due to the sample geometry, the easy axis is assumed to be in x-axis, parallel to the current direction. Supplementary Equation 3 can be solved numerically to obtain the equilibrium angle φ and θ as a function of H.

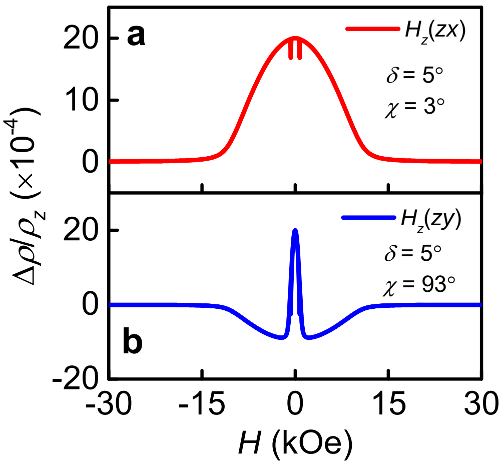

Supplementary Figure 4. Simulated H_z FDMR curves with $\rho_x > \rho_z > \rho_y$ and the small field misalignment. a, H_z is misaligned from z-axis towards x-axis with δ = 5°, χ = 3°; **b,** H_z is misaligned from z-axis towards y-axis with δ = 5°, χ = 93°.

Taking into account the relation $\rho_x > \rho_z > \rho_y$, the MR ratio can be written as:

$$\frac{\Delta\rho}{\rho_z} = \frac{\rho_x - \rho_z}{\rho_z}\sin^2\theta\cos^2\varphi + \frac{\rho_y - \rho_z}{\rho_z}\sin^2\theta\sin^2\varphi \tag{4}$$

By using $\frac{\rho_x - \rho_z}{\rho_z} = 2\times10^{-3}$ and $\frac{\rho_y - \rho_z}{\rho_z} = -1\times10^{-3}$ as estimated from ADMR results in the main text, the H_z FDMR curves can be reproduced in Supplementary Figures 4a and 4b. The parameters used are: H_d = 10 kOe, H_k = 50 Oe and δ = 5°, χ = 3° for Supplementary Figure 4a (or δ = 5°, χ = 93° for Supplementary Figure 4b). As can been from the figure, the M and W shaped MR curves can be reproduced well as long as a small misalignment of H from the z-axis and $\rho_x > \rho_z > \rho_y$ are assumed. The results from control samples, exhibiting different features, will be discussed in Supplementary Note 6 shortly.

Supplementary Note 3. Demagnetizing field effect on angle dependent magnetoresistance (ADMR) results

As discussed in the main text, the fittings of ADMR results in Figs. 2b – 2e exhibit a small deviation from the $\sin^2\theta_{zx}$ or $-\sin^2\theta_{zy}$ dependence, especially in the case of Fe. This is caused by the small

deviation of the magnetization with respect to the external field during *zx*- or *zy*-plane rotation in the presence of a moderate demagnetizing field (H_d). In a similar way, Supplementary Equation 3 can be used to numerically calculate the equilibrium angle φ and θ during the field rotation. Here, we look at the *zy*-plane rotation case as an example, and the *zx*-plane rotation case is similar. We let $\chi = 90^\circ$, $H = 30$ kOe and vary δ from 0 to 360°. From the AHE measurements in Supplementary Figure 5a, H_d in $[Fe_{0.83}Mn_{0.17}(0.6)Pt(0.4)]_{10}/Pt(1)$, $(Fe_{0.71}Mn_{0.29})_{0.6}Pt_{0.4}(9)$, $Fe_{0.71}Mn_{0.29}(9)$ and Fe(9) can be estimated as 10 kOe, 10 kOe, 15 kOe and 20 kOe, respectively. Supplementary Figure 5b shows the calculated θ angle during the *zy*-plane rotation, corresponding to different H_d values. As can be seen, although the magnetization can be aligned in the *z*-axis ($H_d < 30$ kOe), the magnetization indeed presents a small deviation from the perfect alignment at other angles, and this deviation increases as H_d increases. This directly causes the deviation of ADMR fittings as manifested in the $-\sin^2\theta$ calculation in Supplementary Figure 5c. It is clear that these numerical results are in good agreement with the measured ADMR results in Fig. 2 of the main text. However, it should be noted that the presence of the magnetization deviation from the external field does not affect the magnitude of the MR ratio. As long as the external field is above the saturation field, the magnetization is able to reach *z*- and *y*-axis in equilibrium, and therefore, it does not affect the discussion based on the MR ratios.

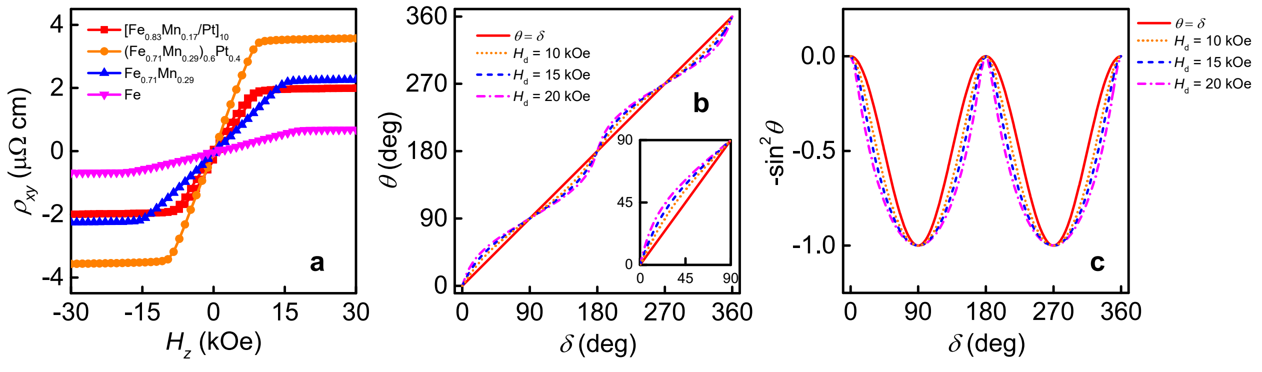

Supplementary Figure 5. Simulated ADMR curve in *zy*-plane rotation. a, Hall resistivity as a function of H_z in $[Fe_{0.83}Mn_{0.17}(0.6)Pt(0.4)]_{10}/Pt(1)$, $(Fe_{0.71}Mn_{0.29})_{0.6}Pt_{0.4}(9)$, $Fe_{0.71}Mn_{0.29}(9)$ and Fe(9); **b,** Simulated magnetization angle θ during *zy*-plane rotation with different H_d values; **c,** Simulated $-\sin^2\theta$ dependence using the θ data in **b.**

Supplementary Note 4. Correlation of MR(θ_{zy}) with anomalous Hall effect (AHE) in $Fe_{1-x}Mn_x$/Pt multilayers and $Fe_{1-x}Mn_x$

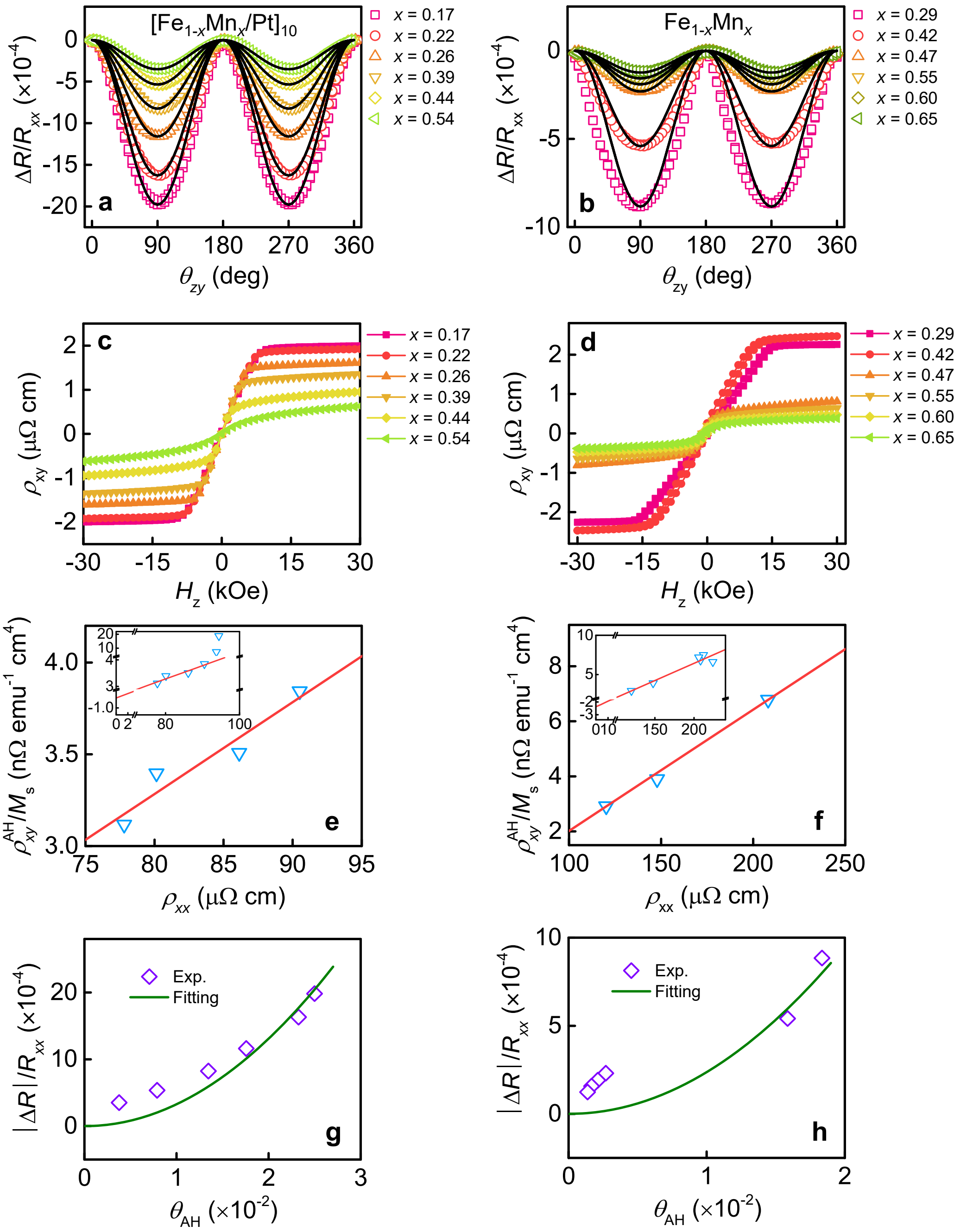

Supplementary Figure 6. Correlation of MR(θ_{zy}) and AHE. **a** and **b,** MR(θ_{zy}) ratio for [$Fe_{1-x}Mn_x$(0.6)/Pt(0.4)]$_{10}$/Pt(1) and $Fe_{1-x}Mn_x$(9), respectively, with $x = 0.17 - 0.65$; **c** and **d**, Hall resistivity as a function of H_z. **e** and **f,** ρ_{xy}^{AH} / M_s as a function of ρ_{xx} in the linear range. **g** and **h,** Plot of MR ratio as a function of θ_{AH} and fitting using the quadratic relation θ_{AH}^2. Insets in **e** and **f** are the full range of the plot in the respective figure, and solid lines in **e** and **f** serve as a guide for the eye.

Supplementary Figures 6a and 6b show the MR(θ_{zy}) curves for $[Fe_{1-x}Mn_x(0.6)/Pt(0.4)]_{10}/Pt(1)$ and $Fe_{1-x}Mn_x(9)$, respectively, with $x = 0.17 – 0.65$. Similar to the results shown in Fig. 3 of the main text for co-sputtered $(Fe_{1-x}Mn_x)_{0.6}Pt_{0.4}(9)$ samples, the MR ratio in these two types of samples also decreases with the increase of Mn composition. In addition, the decreasing trend happens to coincide with the experimentally determined M_s dependence on Mn composition (see Supplementary Figures 2a and 2c). Supplementary Figures 6c and 6d show the Hall measurement results for $Fe_{1-x}Mn_x/Pt$ multilayer and $Fe_{1-x}Mn_x$ samples with field applied perpendicular to the plane. Similar to the procedures described in the main text for the co-sputtered samples, we extracted ρ_{xy}^{AH} from the measured Hall resistivity and then plot ρ_{xy}^{AH} / M_s as a function of ρ_{xx} in Supplementary Figures 6e and 6f for $[Fe_{1-x}Mn_x(0.6)/Pt(0.4)]_{10}/Pt(1)$ and $Fe_{1-x}Mn_x(9)$, respectively. Interestingly, the linear fitting still holds in both cases for samples in the low Mn composition range, whose *M-T* curves can be fitted by Supplementary Equation 1. At high Mn compositions, due to the weakening of FM order and onset of AF order, the fitting significantly deviates from the linear relation, particularly for the multilayer sample. As with the case of co-sputtered samples, θ_{AH} can be calculated from $\theta_{AH} = \rho_{xy}^{AH} / \rho_{xx}$. With these values, we can plot MR(θ_{zy}) ratio as a function of θ_{AH}, as shown in Supplementary Figures 6g and 6h, respectively. Interestingly, for $Fe_{1-x}Mn_x/Pt$ multilayers, the quadratic relation between MR(θ_{zy}) and θ_{AH} still holds approximately including samples with high Mn composition. This is not surprising because the saturation magnetization does not appear explicitly in the drift-diffusion formalism, though it may indirectly affect the scattering asymmetry parameters. Further studies are required to gain an insight of the role of M_s in determining the MR(θ_{zy}) versus θ_{AH} relation. On the other hand, the fitting for $Fe_{1-x}Mn_x$ only serves as guide for eye due to the small number of data points. As mentioned above, the magnetic properties of $Fe_{1-x}Mn_x$ changes drastically when x approaches and exceeds 0.5. A more rigorous theoretical model is required to deal with AHE of such kind of materials with complex spin structures. Before ending this supplementary note, it is worth pointing out that, in the case

of multilayers, MR(θ_{zy}) may also originate from SHE in the individual Pt layers[12] or interface scattering[13], as we reported previously[3, 4]. However, it is difficult to distinguish AHE and SHE contributions to MR(θ_{zy}) as both exhibit the same angular dependence.

Supplementary Note 5. Determination of anomalous Hall resistivity from Hall measurements

Hall resistivity in FM metals can be empirically written as[14]: $\rho_{xy} = R_0 H_z + R_s M(H_z)$, where R_0 and R_s are the ordinary Hall effect (OHE) and AHE coefficients, respectively, H_z is the applied magnetic field in z-direction, and $M(H_z)$ is the magnetization at H_z. The OHE arises from the Lorentz force generated by the magnetic field, which goes to zero when $H_z = 0$. Therefore, in order to extract the AHE contribution ρ_{xy}^{AH}, ρ_{xy} is extrapolated from positive and negative high fields to zero field as indicated by the solid line in Supplementary Figure 7. Here, the Hall result for $(Fe_{1-x}Mn_x)_{0.6}Pt_{0.4}(9)$ is used as an example. The difference of the two intercepts (positive and negative) at $H_z = 0$ corresponds to $2\rho_{xy}^{\mathrm{AH}}$. On the other hand, the slope of the solid line corresponds to R_0. By repeating the above process, ρ_{xy}^{AH} in different samples were obtained.

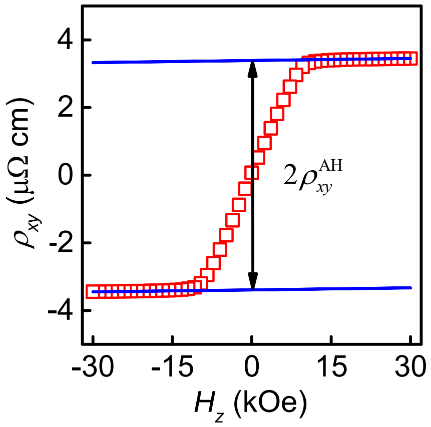

Supplementary Figure 7. Extraction of ρ_{xy}^{AH} from Hall measurements. An example illustrates the process of extracting ρ_{xy}^{AH} from Hall measurement results.

Supplementary Note 6. Control measurements on Co, Py and $Ir_{0.2}Mn_{0.8}$ thin films

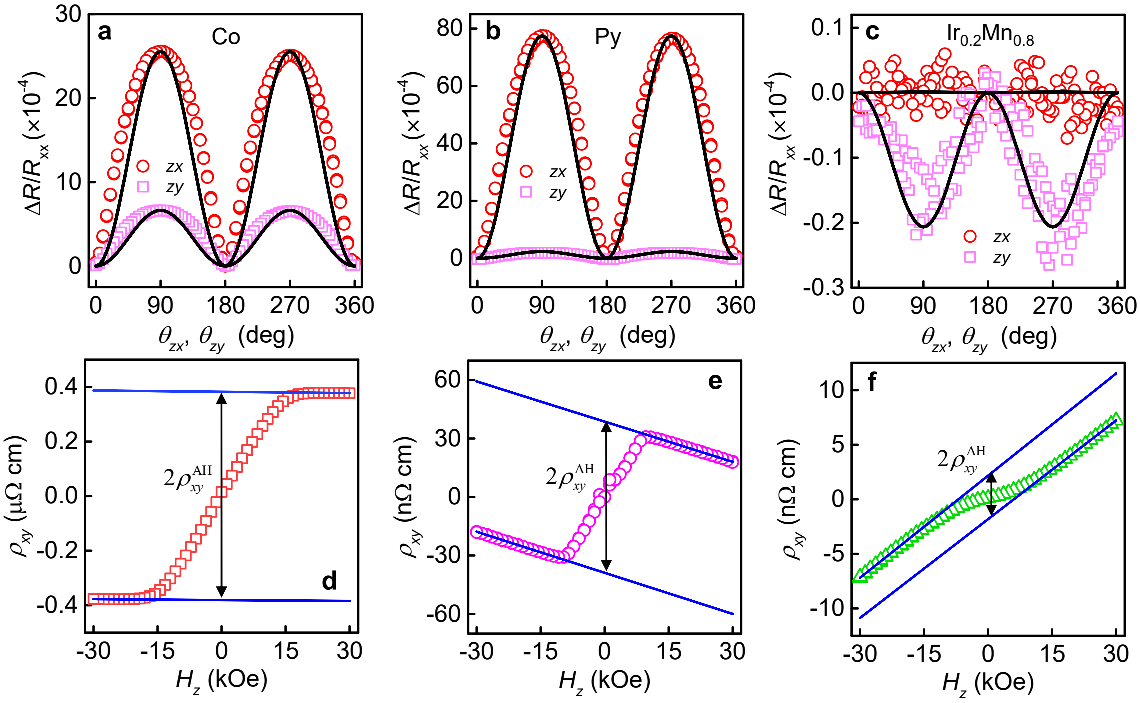

Supplementary Figure 8. Investigation of AHMR in Co, Py and $Ir_{0.2}Mn_{0.8}$ thin films. a – c, ADMR measurement results: **a,** Co; **b,** Py; **c,** $Ir_{0.2}Mn_{0.8}$. **d – f,** Hall measurement results: **d,** Co; **e,** Py; **f,** $Ir_{0.2}Mn_{0.8}$. Solid lines in **a - c** are the fittings based on the angle dependence; and those in d – f are the linear fitting to extract ρ_{xy}^{AH} using the method described in Supplementary Note 5.

In addition to Fe based materials, we performed the same magnetoresistance measurements for control samples including Co(9), Py(9) and $Ir_{0.2}Mn_{0.8}$(9) thin films. Supplementary Figures 8a – 8c show the MR results for *zx* and *zy* plane scans in these samples, respectively. And shown in Supplementary Figures 8d – 8f are the respective Hall measurement results. Following the discussion in the main text, MR(θ_{zx}) corresponds to the AMR while MR(θ_{zy}) is from the AHMR. As expected, a sizable AMR [MR(θ_{zx}) signal] can be observed in both Co and Py samples, and it is vanished in $Ir_{0.2}Mn_{0.8}$. On the other hand, the MR(θ_{zy}) signal in Co and Py exhibits an opposite polarity with that expected for AHMR, which gives $\rho_y > \rho_z$. Previous reports suggested that such kind of behavior may come from the geometric size effect (GSE)[15-17], which itself is still debatable as different mechanisms have been suggested such as electronic structure

in thin films[15], anisotropic interfacial scattering[16] and anisotropic *sd* scattering of minority spins[17]. Similar observation of $\rho_x > \rho_y > \rho_z$ has been observed in Ni[15], Py[16] and Co[17] in these previous reports. Put the origin aside first, our results suggested that AHMR in Co and Py samples are much smaller than those from Fe-based samples and are masked out by GSE related MR. For $Ir_{0.2}Mn_{0.8}$, although the polarity of MR(θ_{zy}) agrees with the prediction of AHMR, its size is around two orders of magnitude smaller than that observed in $Fe_{1-x}Mn_x$ based systems. This is understandable because although a relatively large spin Hall angle, around 80% of that of Pt, has been reported for $Ir_{0.2}Mn_{0.8}$[18, 19], as an AF, it does not have a net moment which is required for observing AHE.

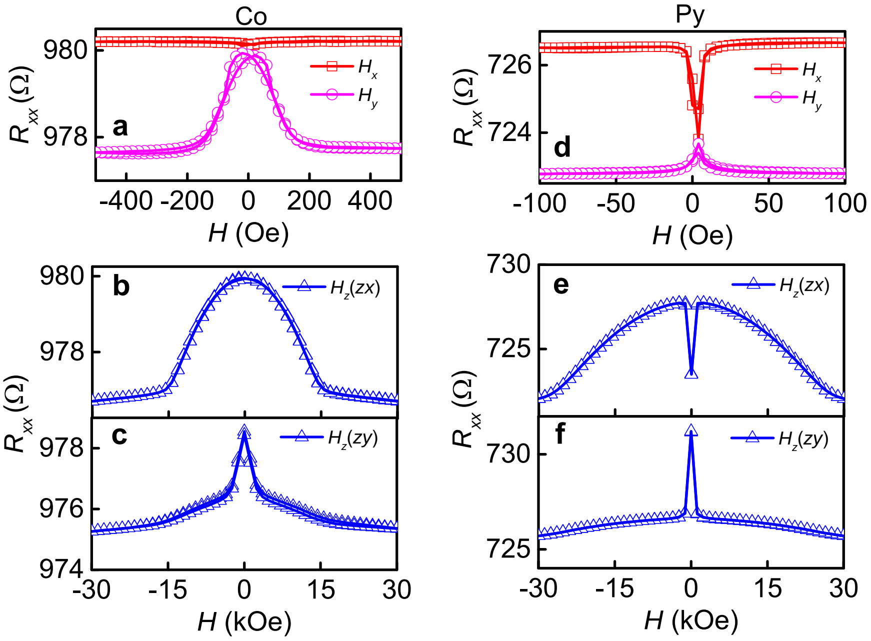

Supplementary Figure 9. FDMR curves for control samples. a – c, Co(9); **d – f,** Py(9). The legends H_x, H_y and H_z denotes the FDMR curves obtained when the field is swept in *x*, *y*, and *z*-axis direction, respectively; and *zx* (or *zy*) in the parenthesis after H_z indicates the misalignment of H_z from *z*-axis towards *x*-axis (or *y*-axis).

The FDMR measurements were also performed for Co and Py to confirm the different relation of $\rho_x > \rho_y > \rho_z$ as compared to the Fe based samples. The results are shown in Supplementary Figures 9a – 9c for Co and Supplementary Figures 9d – 9f for Py with different sweeping field directions. The general shape of H_x and H_y FDMR curves (Supplementary Figures 9a and 9d) are similar to the previous ones in

Supplementary Note 2, which gives $\rho_x > \rho_y$. The different magnitude of the dip or peak in the center small field region is related to the detailed domain structure formed during the sweeping[17]. The H_z FDMR curves with misalignment to x-axis also looks similar to those in Supplementary Note 2, but those with misalignment to y-axis exhibit a totally different shape. This is directly related to $\rho_y > \rho_z$. The same macro-spin approach can be applied to the Co and Py case as well to determine θ and φ. The difference is that in the MR ratio simulation, a positive ratio of $\frac{\rho_y - \rho_z}{\rho_z} = 1\times10^{-3}$ should be adopted. For illustration purpose, we use the same set of parameters to simulate the MR curves, and the results are presented in Supplementary Figures. 10a and 10b. As can be seen, indeed the shapes can be accounted for by the assumption of a small misalignment of H from the z-axis and $\rho_x > \rho_y > \rho_z$. Both the FDMR and ADMR results suggest that the MR effects, especially MR(θ_{zy}) in Fe based samples are different from those in Co and Py.

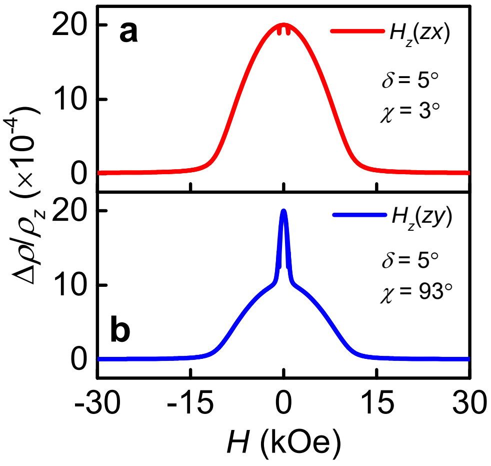

Supplementary Figure 10. Simulated H_z FDMR curve with $\rho_x > \rho_y > \rho_z$ and the small field misalignment. a, H_z is misaligned from z-axis towards x-axis with δ = 5°, χ = 3°; **b,** H_z is misaligned from z-axis towards y-axis with δ = 5°, χ = 93°.

The difference in MR prompted us to look into the AHE in these samples. In general, the Hall resistivity in these control is at least one order of magnitude smaller than that in Fe based systems with the same thickness. By using the method described in Supplementary Note 5, we separated the contribution of OHE

and AHE from the Hall effect results. The values are summarized in Supplementary Table 2 and compared with those obtained from Fe based systems. It should be noted that based on early theoretical calculations on intrinsic AHE[20, 21], both OHE and AHE resistivity are negative for Ni, while both of them are positive for Fe; and for Co, OHE is negative but AHE is positive. Here, the positive resistivity refers to the positive Hall voltage in the positive *y*-axis direction when the current is in the positive *x*-axis direction and magnetic field is in positive *z*-axis direction. As can be seen from the table, the signs of the OHE and AHE contributions follow the calculations, if one considers the fact that Fe is the major composition in these samples, except for ρ_{xy}^{AH} in Py. In fact, it has been pointed out that due to the small strength of AHE, the sign of ρ_{xy}^{AH} in Py is very sensitive to chemical composition as well as the thickness of film, and therefore both positive and negative values has been reported in Py[22]. From these values, it is clear that smaller θ_{AH} is the direct cause of the difference in MR(θ_{zy}) between Co, Py and $Ir_{0.2}Mn_{0.8}$ and Fe-based samples.

Supplementary Table 2. Comparison of the OHE, AHE resistivity and AHE angle among $Fe_{0.83}Mn_{0.17}$/Pt, $(Fe_{0.71}Mn_{0.29})_{0.6}Pt_{0.4}$, $Fe_{0.71}Mn_{29}$, $Fe_{0.75}Pt_{25}$, Fe, Co, Py and $Ir_{0.2}Mn_{0.8}$. The thickness of these samples are fixed at 9 nm.

Type	ρ_0 (μΩ cm)	ρ_{xy}^{AH} (μΩ cm)	θ_{AH}
$Fe_{0.83}Mn_{0.17}$/Pt	0.12±0.02	1.94±0.01	$0.025\pm7.55\times10^{-5}$
$(Fe_{0.71}Mn_{0.29})_{0.6}Pt_{0.4}$	0.15±0.03	3.38±0.04	$0.028\pm1.60\times10^{-4}$
$Fe_{0.71}Mn_{29}$	0.12±0.01	2.21±0.05	$0.018\pm1.75\times10^{-4}$
$Fe_{0.75}Pt_{25}$	0.12±0.02	1.61±0.02	$0.022\pm1.14\times10^{-4}$
Fe	0.07±0.01	0.65±0.01	$0.0096\pm5.69\times10^{-5}$
Co	-0.009±0.002	0.38±0.001	$0.0037\pm9.32\times10^{-6}$
Py	-0.05±0.001	0.04±0.0003	$5.71\times10^{-4}\pm4.11\times10^{-6}$
$Ir_{0.2}Mn_{0.8}$	0.02±0.001	-0.002±0.0002	$-7.30\times10^{-6}\pm8.27\times10^{-7}$

Supplementary Note 7. Discussion on magnetic field misalignment and geometric size effect (GSE) related anisotropic magnetoresistance (AMR) contributions

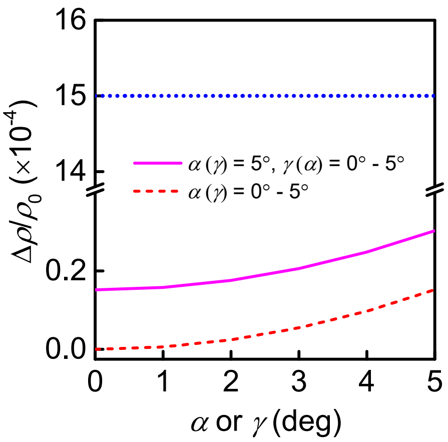

Supplementary Figure 11. Simulated AMR contribution to MR(θ_{zy}) ratio due to field misalignment. Solid line: $\alpha = 5^\circ$ and $\gamma = 0^\circ$ to 5° or $\alpha = 0^\circ$ to 5° and $\gamma = 5^\circ$. $\alpha = 0^\circ$ and $\gamma = 0^\circ$ to 5° or $\alpha = 0^\circ$ to 5° and $\gamma = 0^\circ$. Dotted line: experimental MR(θ_{zy}) ratio for $(Fe_{0.71}Mn_{0.29})_{0.6}Pt_{0.4}$ with a thickness of 9 nm.

Before proceeding further, it is necessary to exclude other contributions besides AHE as the main cause for the MR(θ_{zy}). In this note, we first discuss the influence of field misalignment contribution. During the *zy* plane ADMR measurement, the misalignment of either field or sample (which are relative to each other) can be represented by a small rotation around *y*-axis by γ and *z*-axis by α. Assume that, at perfect alignment, the magnetization vector is given by:

$$\mathbf{m} = \begin{pmatrix} 0 \\ \sin\theta_{zy} \\ \cos\theta_{zy} \end{pmatrix} \tag{5}$$

After the rotation around *y*- and *z*-axis, the magnetization vector is given by:

$$\mathbf{m} = \begin{pmatrix} \cos\alpha & -\sin\alpha & 0 \\ \sin\alpha & \cos\alpha & 0 \\ 0 & 0 & 1 \end{pmatrix} \begin{pmatrix} \cos\gamma & 0 & \sin\gamma \\ 0 & 1 & 0 \\ -\sin\gamma & 0 & \cos\gamma \end{pmatrix} \begin{pmatrix} 0 \\ \sin\theta_{zy} \\ \cos\theta_{zy} \end{pmatrix}$$

$$= \begin{pmatrix} \cos\alpha\sin\gamma\cos\theta_{zy} - \sin\alpha\sin\theta_{zy} \\ \sin\alpha\sin\gamma\cos\theta_{zy} + \cos\alpha\sin\theta_{zy} \\ \cos\gamma\cos\theta_{zy} \end{pmatrix} \tag{6}$$

If the observed MR(θ_{zy}) is due to the misalignment of conventional AMR only, the angle dependent longitudinal resistivity should be given by:

$$\rho = \rho_0 + \Delta\rho(\cos\alpha \sin\gamma \cos\theta_{zy} - \sin\alpha \sin\theta_{zy})^2 \quad (7)$$

where $\Delta\rho/\rho_0$ is the AMR ratio, about 2×10^{-3} estimated from the ADMR results in Fig. 2 of the main text. Using Supplementary Equation 7, we calculated the contribution of AMR from field misalignment in the MR(θ_{zy}) with different combinations of misalignment angles up to 5˚, and the results are plotted in Supplementary Figure 11. The solid line refers to the case where both rotations exist, and one of them is fixed at 5˚ with the other varying from 0˚ to 5˚; while the dashed line is the case where only one of the rotation exists with an angle from 0˚ to 5˚. As a comparison, the signal level of MR(θ_{zy}) of the FeMnPt sample observed in Fig. 2 of the main text is also added in the figure (dotted line). It should be noted that the misalignment angle above 5˚ is highly unlike in the present experimental setup. It is clearly that the size of the signal (about 10^{-5}) is always nearly two orders of the magnitude smaller than that observed in Fe based samples. There must be another mechanism that gives rise to the MR(θ_{zy}) signal, that is, the AHMR.

In addition, to exclude GSE related AMR as the origin of MR(θ_{zy}) in Fe based samples, temperature dependent ADMR measurements were performed on $(Fe_{0.71}Mn_{0.29})_{0.6}Pt_{0.4}$(9), Fe(9) and Py(9) samples. Despite its debatable underlying mechanism, GSE related AMR should still follow the temperature dependence of conventional AMR[17]. In other words, one should expect at least a same temperature dependence for MR(θ_{zy}) and MR(θ_{zx}) if MR(θ_{zy}) is dominated by GSE. The ADMR results of MR(θ_{zy}) and MR(θ_{zx}) in the temperature range of 50 – 300 K are presented in Supplementary Figures 12a – 12b for the FeMnPt sample, and the ratios are further summarized in Supplementary Figure 12c. In a similar manner, Supplementary Figures 12d – 12f present the results of the Fe sample, and Supplementary Figures 12i – 12h are those of the Py sample. In all three types of samples, we observed the increasing MR(θ_{zx}) ratio with the decrease of temperature, which supports the explanation that MR(θ_{zx}) is originated from the

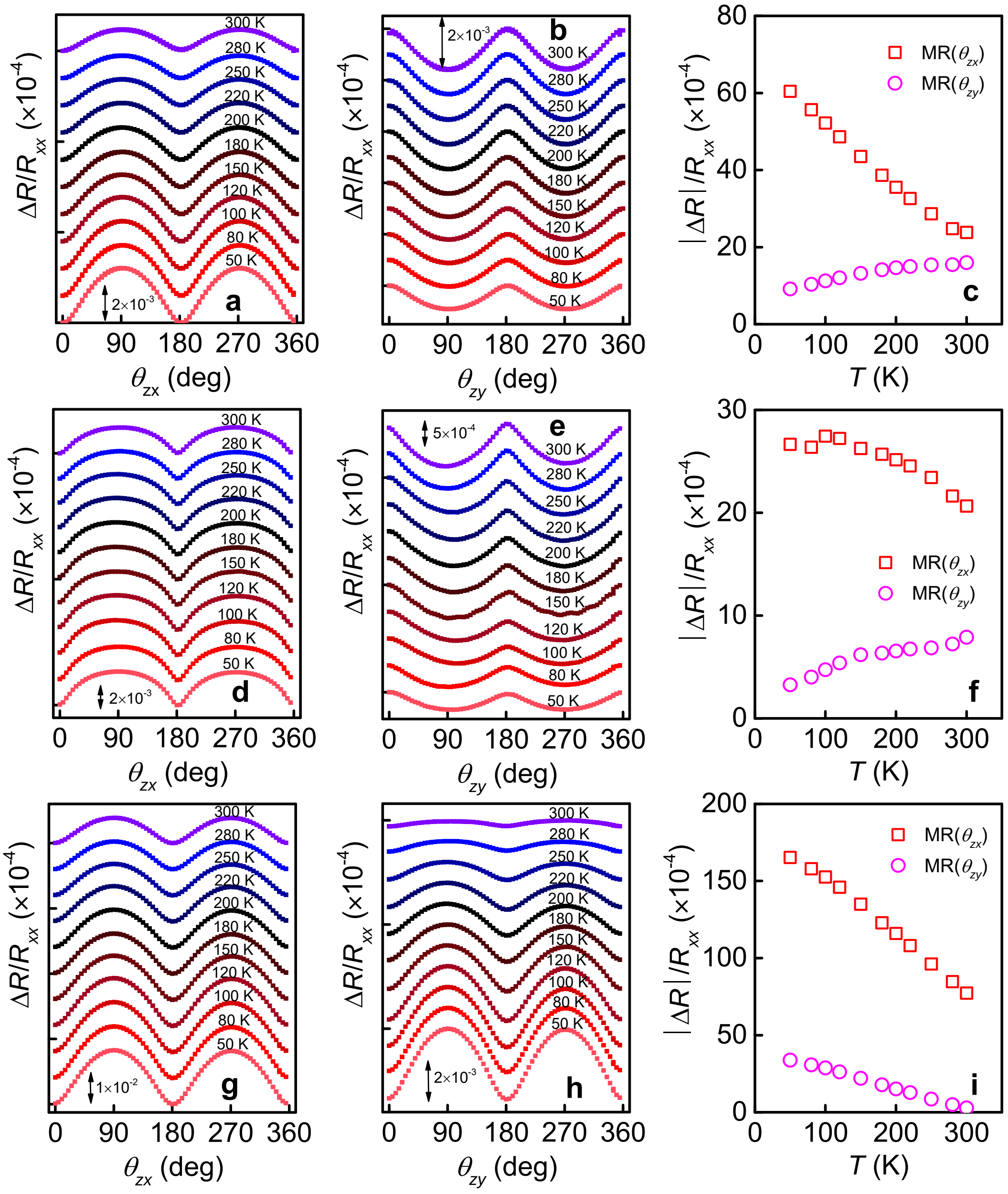

Supplementary Figure 12. Temperature dependence of MR(θ_{zy}) and MR(θ_{zx}). a, and **b,** ADMR results for $(Fe_{0.71}Mn_{0.29})_{0.6}Pt_{0.4}$(9); **c,** Summary of the MR ratios for $(Fe_{0.71}Mn_{0.29})_{0.6}Pt_{0.4}$(9); **d,** and **e,** ADMR results for Fe(9); **f,** Summary of the MR ratios for Fe(9); **g,** and **h,** ADMR results for Py(9); **i,** Summary of the MR ratios for Py(9).

conventional AMR. This temperature dependence can be understood in the sense that the AMR ratio is enhanced by the reduction of phonon mediated *sd* scattering at low temperature. As for the MR(θ_{zy}) ratio, we indeed observed the same increasing temperature dependence in the Py case. This supports our explanation that MR(θ_{zy}) in Py is dominated by GSE related AMR. In fact, similar observations have been

reported and attributed to GSE in Co in the literature[17]. On the contrary, in Fe and Fe-based alloys, a totally opposite temperature dependence has been observed for MR(θ_{zy}) ratio, *i.e.*, it decreases with the decrease of temperature. This suggest that MR(θ_{zy}) and MR(θ_{zx}) in these samples have a different origin. As discussed in the main text, the AHMR ratio is given by $(\frac{\theta_{\mathrm{AH}}}{\beta})^2\frac{2l_{\mathrm{s}}}{d}\tanh(\frac{d}{2l_{\mathrm{s}}})$. As summarized in Supplementary Figure 13a for the FeMnPt sample, θ_{AH} can be obtained experimentally, and it is not very sensitive to temperature in the range of 50 – 300 K. On the other hand, although β and l_{s} is not directly accessible, one would expect both of them to increase with the decrease of temperature. To investigate their respective role on AHMR ratio, we calculated the β and l_{s} dependence of AHMR ratio in Supplementary Figure 13b (β = 0.2 – 0.8, l_{s} = 4.5 nm) and Supplementary Figure 13c (β = 0.55, l_{s} = 2 – 8 nm). In both figures, θ_{AH} is taken as 0.028, and the data in x- and y-axis are normalized to the minimum value in each axis. As can be seen, the increase of β would lead to a decrease in AHMR ratio, whereas an opposite trend is obtained for l_{s}. However, for a same increase by a factor of 4, the effect of β on AHMR is about 10 times larger than that of l_{s}. Therefore, the temperature dependence should be mainly determined by β, which agrees with the general trend of experimental temperature-dependence of AHMR in the FeMnPt samples. In fact, β has also been found to play an important role in determining the temperature dependence of SMR in W/CoFeB bilayers[23]. Although further systematic studies are required to quantitatively elucidate the temperature dependence of AHMR, which is out of the scope of this manuscript, from the aforementioned experimental results and analysis, one can rule out GSE related AMR as the origin of MR(θ_{zy}).

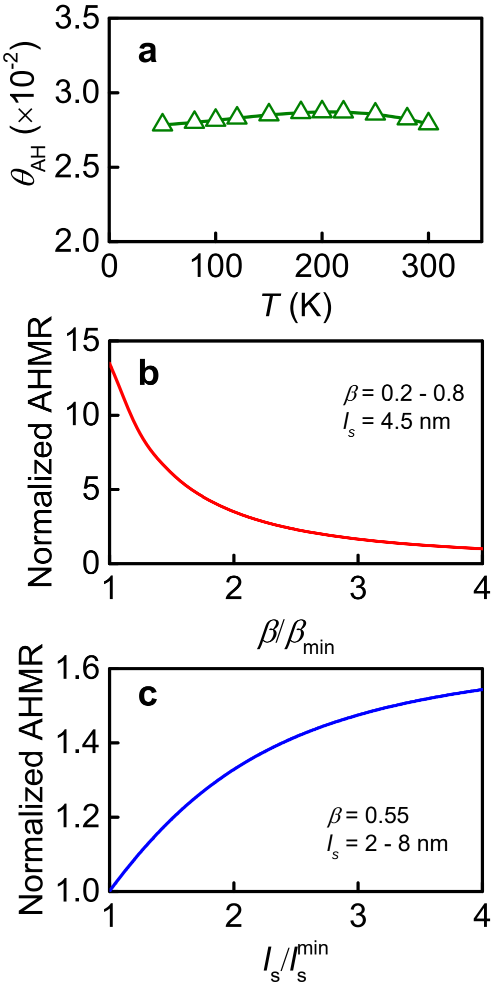

Supplementary Figure 13. Effect of θ_{AH}, β and l_s on the temperature dependence of AHMR. a, Experimentally obtained temperature dependence of θ_{AH} in $(Fe_{0.71}Mn_{0.29})_{0.6}Pt_{0.4}$(9); **b,** Calculated β dependence of AHMR ratio with $\beta = 0.2 – 0.8$, $l_s = 4.5$ nm; **c,** Calculated l_s dependence of AHMR ratio with $\beta = 0.55$, $l_s = 2 – 8$ nm.

Supplementary Note 8. Derivation of anomalous Hall magnetoresistance (AHMR)

To model the AHMR effect, we begin with the definition of the spin Hall angle θ_{SH} and the anomalous conductivity $\sigma_{xy}^{\mathrm{AH}}$ for a bulk conducting FM:

$$j_{\uparrow}^{\mathrm{t}} = \theta_{\mathrm{SH}} j_{\uparrow} \tag{8}$$

$$j_{\downarrow}^{\mathrm{t}} = -\theta_{\mathrm{SH}} j_{\downarrow} \tag{9}$$

where $j^{t}_{\uparrow,\downarrow}$ are the transverse current induced by the longitudinal current $j_{\uparrow,\downarrow}$. The anomalous transverse charge (j^{t}_{c}) and spin current (j^{t}_{s}) is then,

$$j^{t}_{c} = j^{t}_{\uparrow} + j^{t}_{\downarrow} = \theta_{SH}\beta j_{c} \quad (10)$$

$$j^{t}_{s} = j^{t}_{\uparrow} - j^{t}_{\downarrow} = \theta_{SH}(j_{\uparrow} + j_{\downarrow}) = \theta_{SH} j_{c} \quad (11)$$

where $\beta = (j_{\uparrow} - j_{\downarrow})/(j_{\uparrow} + j_{\downarrow})$ is the spin polarization of the longitudinal current. The non-zero charge current would lead to a charge buildup at the side of the conductance which would exactly cancel the transverse charge current. Thus, the ratio of the anomalous conductivity σ^{AH}_{xy} to the longitudinal conductivity σ_{xx} (or anomalous Hall angle) is $\theta_{AH} \equiv \sigma^{AH}_{xy} / \sigma_{xx} \equiv j^{t}_{c} / j_{c} = \beta\theta_{SH}$. It should be noted that from Supplementary Equation 10 and 11, the spin polarization of the transverse conductivity (ζ) can be inferred as $\zeta = j^{t}_{s} / j^{t}_{c} = 1/\beta$. In practice, ζ may not be related to β in this simple way[24, 25]: β is determined by the spin-dependent density of states at Fermi level and scattering asymmetry between spin-up and spin-down electrons in FM and its value is always between -1 and 1; while ζ is not entirely determined by the same mechanisms since the spin-up and spin-down electrons may not be deflected to opposite directions with equal probability due to the fact that scattering potentials seen by the two types of electron are different. However, as discussed by Taniguchi *et al.*[26, 27], if one ignores the energy-dependence of scattering and assume that the deflected spin-up and spin-down electrons will undergo the same spin-dependent scattering as the longitudinal transport, then ζ can be related to β as $\zeta = 1/\beta$. This treatment largely simplifies the relation between θ_{AH} and θ_{SH} as is shown above.

Next we turn to look at how the spin current can affect the longitudinal resistance. The transverse spin current j^{t}_{s} freely flows in the bulk, but would lead to spin accumulation at the edge of the sample. Different from the charge accumulation which acts on the entire sample to cancel the transverse current completely, the spin accumulation only acts on the vicinity of the sample boundary and thus the spin

current in the interior of the sample remains to be $j_c\theta_{AH}/\beta$. The transverse spin current in turn can generate a charge current whose flow direction is always opposite to the original charge current. Thus the total charge current would be $j_c - j_c(\theta_{AH}/\beta)^2$, and it increases the resistance of the film by a factor of $[1-(\theta_{AH}/\beta)^2]^{-1} = 1+(\theta_{AH}/\beta)^2$ due to the combined charge to spin and spin to charge conversion.

Now we apply the above procedure to a thin FM film with thickness of d in z-direction, and the applied current is fixed at x-direction. With the presence of large exchange field, the spins in FM are either parallel or antiparallel to the magnetization direction ($\mathbf{m}$). Bear this in mind, below we further discuss the three cases with $\mathbf{m}$ aligned in different directions by external magnetic field.

When $\mathbf{m}$ is in z-direction, the spin current flows in y-direction and the spin accumulation is at the front and back edges of the film. Due to the much larger dimension of the film width as compare to the spin diffusion length (l_s), the spin current is not affected inside the film and thus the resistance would be the same as the bulk FM case. Therefore, the total transverse spin current, longitudinal charge current and resistivity are summarized respectively as

$$j_s^t(\mathbf{m}\,||\,\mathbf{z}) = j_c\theta_{AH}/\beta \tag{12}$$

$$j_{cx}(\mathbf{m}\,||\,\mathbf{z}) = j_c - j_c(\theta_{AH}/\beta)^2 \tag{13}$$

$$\rho_{xx}(\mathbf{m}\,||\,\mathbf{z}) = \rho_0[1+(\theta_{AH}/\beta)^2] \tag{14}$$

where ρ_0 is the isotropic resistivity of FM.

When $\mathbf{m}$ is in y-direction, the spin current flows in z-direction. In this case, the spin is accumulated at the surface or interface of the film. Since now d is comparable to l_s, the spin accumulation leads to a backflow of spin current which would greatly reduce the total spin current, and it cancels some of the AHE induced extra resistance. The general solution of spin diffusion equation ($\partial_z^2\mu_s = \mu_s/l_s^2$) is

$\mu_{\rm s}(z) = Ae^{z/l_{\rm s}} + Be^{-z/l_{\rm s}}$. By using the boundary conditions: $j_{\rm s}^{\rm t}(0) = j_{\rm s}^{\rm t}(d) = 0$, we can derive the spin accumulation and transverse spin current in z-direction as:

$$\mu_s(z) = \frac{2el_{\rm s}j_{\rm c}}{\sigma_{xx}}\frac{\theta_{\rm AH}}{\beta}\left(\cosh\left(\frac{z}{l_{\rm s}}\right) - \cosh\left(\frac{z-d}{l_{\rm s}}\right)\right) / \sinh\left(\frac{d}{l_{\rm s}}\right) \tag{15}$$

$$j_{\rm s}^{\rm t}(z) = \frac{j_{\rm c}\theta_{\rm AH}}{\beta}\left[1 - \left(\sinh\left(\frac{z}{l_{\rm s}}\right) - \sinh\left(\frac{z-d}{l_{\rm s}}\right)\right) / \sinh\left(\frac{d}{l_{\rm s}}\right)\right] \tag{16}$$

$$j_{cx}(z) = j_{\rm c} - j_{\rm c}\left(\frac{\theta_{\rm AH}}{\beta}\right)^2\left[1 - \left(\sinh\left(\frac{z}{l_{\rm s}}\right) - \sinh\left(\frac{z-d}{l_{\rm s}}\right)\right) / \sinh\left(\frac{d}{l_{\rm s}}\right)\right] \tag{17}$$

Average the above spin current over the thickness, one has the total transverse spin current, longitudinal charge current and resistivity in this case as

$$j_{\rm s}^{\rm t}(\mathbf{m} \parallel \mathbf{y}) = j_{\rm c}(\theta_{\rm AH}/\beta)\left(1 - \frac{2l_{\rm s}}{d}\tanh(d/2l_{\rm s})\right) \tag{18}$$

$$j_{cx}(\mathbf{m} \parallel \mathbf{y}) = j_{\rm c} - j_{\rm c}(\theta_{\rm AH}/\beta)^2\left(1 - \frac{2l_{\rm s}}{d}\tanh(d/2l_{\rm s})\right) \tag{19}$$

$$\rho_{xx}(\mathbf{m} \parallel \mathbf{y}) = \rho_0\left[1 + (\theta_{\rm AH}/\beta)^2\left(1 - \frac{2l_{\rm s}}{d}\tanh(d/2l_{\rm s})\right)\right] \tag{20}$$

When $\mathbf{m}$ is in x-direction, there is no transverse spin current and thus no extra-resistance. However, the conventional anisotropy magnetoresistance (AMR) would appear, and this leads to the total transverse spin current, longitudinal charge current and resistivity as $j_{\rm s}^{\rm t}(\mathbf{m} \parallel \mathbf{x}) = 0$, $j_{cx}(\mathbf{m} \parallel \mathbf{x}) = j_{\rm c}$ and $\rho_{xx}(\mathbf{m} \parallel \mathbf{x}) = \rho_0(1 + A)$, where A is the AMR ratio. Taken together the above three cases, one can summarize the MR effect in a single FM as

$$\rho_{xx} = \rho_0\left(1 + Am_x^2 + (\theta_{\rm AH}/\beta)^2\left[m_z^2 + \left(1 - \frac{2l_{\rm s}}{d}\tanh(d/2l_{\rm s})\right)m_y^2\right]\right) \tag{21}$$

Supplementary Note 9. Effect of surface roughness and film thickness on transport and magnetic properties in ultrathin films

It is known that the percolated structure or significant surface roughness in very thin films can affect both the electrical and magnetic properties. In addition, any change in the surface condition after the sample was exposed to ambient may also affect its physical properties. As the films under investigation are polycrystalline in nature, it would be difficult to achieve layer-by-layer growth at atomic layer accuracy and therefore, the presence of a certain degree of roughness is unavoidable. We have previously investigated systematically the electrical properties of ultrathin metallic film[28], including Al, Au, Cr, Cu, Ru, Ta, $Co_{90}Fe_{10}$, $Ni_{81}Fe_{19}$, and $Ir_{20}Mn_{80}$. Different materials indeed exhibit different level of roughness. Except for Al, Au and Cu, the root-mean-square (RMS) roughness of remaining films at a thickness of 20 nm is generally below 0.2 nm. The resistivity of all these films show an upturn at small thickness, though the turning point is generally more than one order of magnitude larger than the roughness. This suggests that the thickness at which sharp upturn of resistivity appears is mainly determined by the electron mean free path, as we discussed in main text.

To characterize the roughness of the thin film used in this study, we preformed atomic force microscopy (AFM) measurement on the 5 nm sample of $(Fe_{0.71}Mn_{0.29})_{0.6}Pt_{0.4}$ and Fe. As an example, Supplementary Figure 14 shows the AFM image for $(Fe_{0.71}Mn_{0.29})_{0.6}Pt_{0.4}$ within an area of 5 μm × 5 μm. The averaged RMS roughness over 5 different areas with such a size is 0.26 nm and 0.33 nm for $(Fe_{0.71}Mn_{0.29})_{0.6}Pt_{0.4}$ and Fe, respectively. As shown in Fig. 5b of the main text, the sharp upturn of resistivity in $(Fe_{0.71}Mn_{0.29})_{0.6}Pt_{0.4}$ appears at about 3 nm, which is also about 10 times of the RMS roughness, in good agreement with previous studies. Therefore, the sharp upturn of resistivity kicks in when the thickness of the film becomes comparable to the electron mean free path rather than due to reaching the percolation threshold of forming discontinuous film. In fact, the resistivity values of $(Fe_{0.71}Mn_{0.29})_{0.6}Pt_{0.4}$ and Fe with a thickness of 5 – 20 nm are in the range of 3.9×10^3 – 1.8×10^4 $(\Omega\ cm)^{-1}$

and $4.9\times10^3 - 2.3\times10^4$ $(\Omega\ \mathrm{cm})^{-1}$, respectively. These values fall into the upper bound of bad metal and lower bound of good metal regime[14]. Therefore, we can say that both the $(Fe_{0.71}Mn_{0.29})_{0.6}Pt_{0.4}$ and Fe films with a thickness of 5 – 20 nm are continuous metallic films.

On the other hand, both surface and size-effect also affect magnetic properties of thin films, which typically would lead to decrease of saturation magnetization. There is no generic model to describe the thickness dependence of saturation magnetization in ultrathin films since both the surface and interface with substrate vary from sample to sample. As far as $(Fe_{0.71}Mn_{0.29})_{0.6}Pt_{0.4}$ thin film is concerned, as shown in Fig. 5b of the main text, the magnetization began to decrease at a thickness of ~ 5 nm, which is larger than the thickness at which the resistivity shows a sharp upturn. This is understandable since they are governed by phenomena of different length scale. However, as explained in the main text, this does not affect the analysis and interpretation of the experimental data of films with $d > 5$ nm. For samples with $d < 5$ nm, the experimental data can be understood qualitatively if we take into account the thickness-dependent θ_{AH} obtained experimentally. However, we did not include the fitting results in Fig. 5a of the main text because thickness-dependence of β is unknown both theoretically and experimentally.

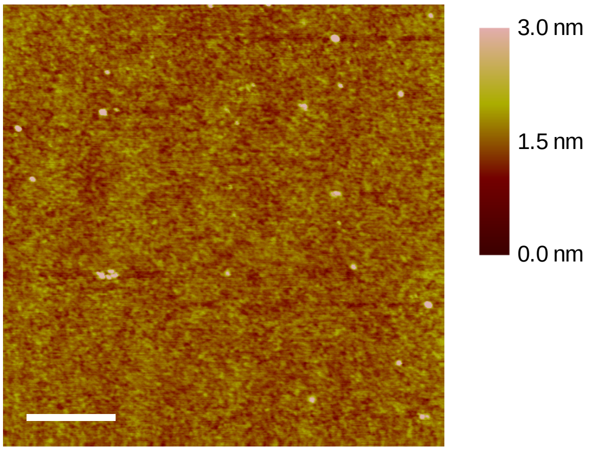

Supplementary Figure 14. Characterization of thin film roughness. AFM image of a 5 nm-thick $(Fe_{0.71}Mn_{0.29})_{0.6}Pt_{0.4}$. The scale bar is 1 μm.

Supplementary Note 10. Thickness dependence of AHMR in thick $(Fe_{0.71}Mn_{0.29})_{0.6}Pt_{0.4}$ and Fe samples

To substantiate the thickness dependence of AHMR, besides the results shown in Fig. 5 of the main text, we fabricated another batch of $(Fe_{0.71}Mn_{0.29})_{0.6}Pt_{0.4}$ and extended the same thickness dependence study to Fe as well. Supplementary Figures 14a and 14b show the MR(θ_{zy}) curves for $(Fe_{0.71}Mn_{0.29})_{0.6}Pt_{0.4}$ and Fe samples. To avoid any ambiguity, this time we focused on the thickness range of 5 – 20 nm, where the ferromagnetic and electrical properties of the films is almost unchanged. This is also evident in the plot of $\rho_{xy}^{\mathrm{AH}} / M_{\mathrm{s}}$ as a function of ρ_{xx} in Supplementary Figure 14c, which exhibits an almost linear scaling. As summarized in Supplementary Figure 14d, despite some variations in the absolute values at some thicknesses (due to some slight differences in measurement environment for different runs), the general trend of MR(θ_{zy}) is the same as that presented in Fig. 5, *i.e.* AHMR decreases as film thickness increases for $d > 5$ nm. This is in good agreement with the thickness dependence predicted by AHMR theory. In fact, by taking $\beta = 0.62$, $l_{\mathrm{s}} = 6.5$ nm, $\theta_{\mathrm{AH}} = 0.027$ for FeMnPt, and $\beta = 0.32$, $l_{\mathrm{s}} = 3.2$ nm, $\theta_{\mathrm{AH}} = 0.009$ for Fe, both sets of data can be fitted well to the theoretical model. It should be noted that the differences in the parameters used here and those in the main text for FeMnPt may be caused by the differences in the detailed sample preparation and measurement processes, as well as surface conditions. Nevertheless, in view of all these thickness dependence results, one can see that MR(θ_{zy}) does follow the theoretical model.

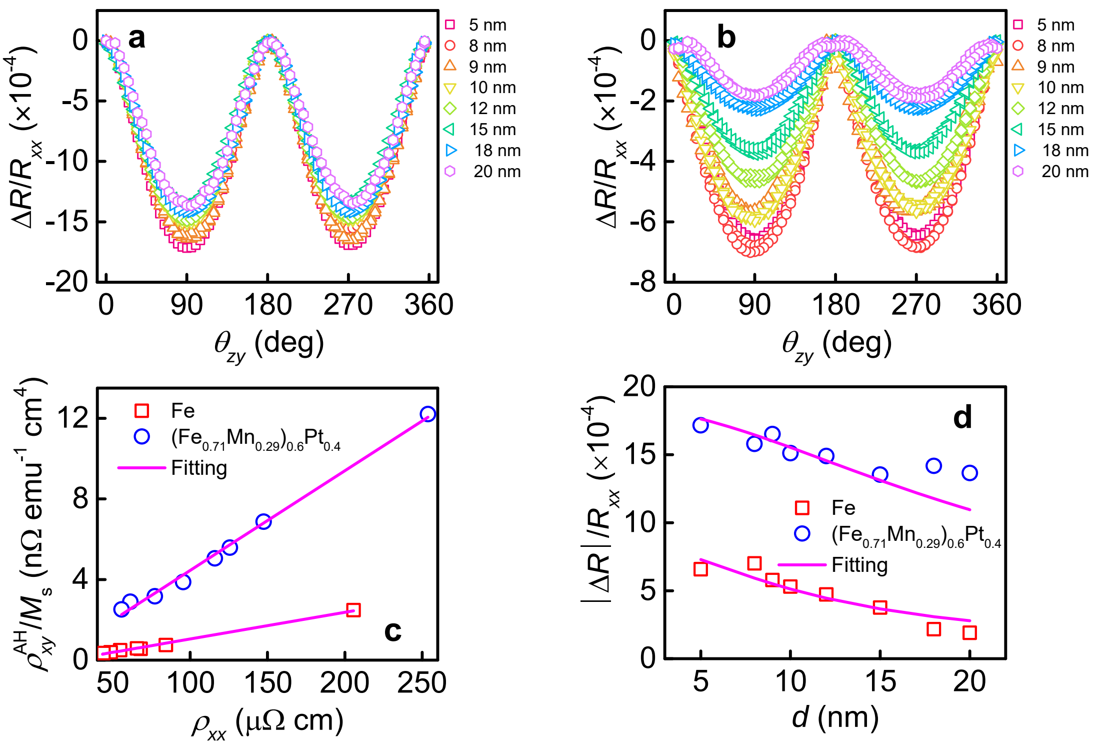

Supplementary Figure 15. Thickness dependence of AHMR in the thick thickness region. a, and **b,** MR(θ_{zy}) results for the new batch of $(Fe_{0.71}Mn_{0.29})_{0.6}Pt_{0.4}$ and Fe, respectively; **c,** Plot of ρ_{xy}^{AH} / M_s as a function of ρ_{xx} in the $(Fe_{0.71}Mn_{0.29})_{0.6}Pt_{0.4}$ and Fe samples; **d,** Summary of the thickness dependence of MR(θ_{zy}) and fitting using the theoretical model.

Supplementary References:

1. Endoh, Y. & Ishikawa, Y. Antiferromagnetism of γ Iron Manganes Alloys. *J. Phys. Soc. Jpn.* **30,** 1614-1627 (1971).

2. Seifert, J., Bernhard, T., Gruyters, M. & Winter, H. Magnetic interface coupling between Co and binary Fe_xMn_{100-x} alloys in the ultrathin film limit. *Phys. Rev. B* **76,** 224405 (2007).

3. Xu, Y*., et al.* Self-current induced spin-orbit torque in FeMn/Pt multilayers. *Sci. Rep.* **6,** 26180 (2016).

4. Luo, Z*., et al.* Static and dynamic magnetic properties of FeMn/Pt multilayers. *J. Appl. Phys.* **121,** 223901 (2017).

5. Liu, Y*., et al.* Configuration of the uncompensated moments at the FM/AFM interface with a NM spacer. *J. Phys. D: Appl. Phys.* **41,** 205006 (2008).

6. Kuz'min, M. D. Shape of temperature dependence of spontaneous magnetization of ferromagnets: quantitative analysis. *Phys. Rev. Lett.* **94,** 107204 (2005).

7. Kuz'min, M. D., Richter, M. & Yaresko, A. N. Factors determining the shape of the temperature dependence of the spontaneous magnetization of a ferromagnet. *Phys. Rev. B* **73,** 100401(R) (2006).

8. Le Guillou, J. C. & Zinn-Justin, J. Critical Exponents for then-Vector Model in Three Dimensions from Field Theory. *Phys. Rev. Lett.* **39,** 95-98 (1977).

9. Alvarado, S., Campagna, M. & Hopster, H. Surface Magnetism of Ni(100) near the Critical Region by Spin-Polarized Electron Scattering. *Phys. Rev. Lett.* **48,** 51-54 (1982).

10. Gradmann, U. Surface magnetism. *J. Magn. Magn. Mater.* **100,** 481-496 (1991).

11. Raquet, B*., et al.* Electron-magnon scattering and magnetic resistivity in 3d ferromagnets. *Phys. Rev. B* **66,** 024433 (2002).

12. Nakayama, H*., et al.* Spin Hall magnetoresistance induced by a nonequilibrium proximity effect. *Phys. Rev. Lett.* **110,** 206601 (2013).

13. Kobs, A*., et al.* Anisotropic interface magnetoresistance in Pt/Co/Pt sandwiches. *Phys. Rev. Lett.* **106,** 217207 (2011).

14. Nagaosa, N*., et al.* Anomalous Hall effect. *Rev. Mod. Phys.* **82,** 1539-1592 (2010).

15. Chen, T. T. & Marsocci, V. A. Transverse Magnetoresistivity Anisotropy Measurements and the Geometrical Size Effect in Nickel Thin Films. *J. Appl. Phys.* **43,** 1554-1558 (1972).

16. Rijks, T. G. S. M., Lenczowski, S. K. J., Coehoorn, R. & Jonge, W. J. M. d. In-plane and out-of-plane anisotropic magnetoresistance in $Ni_{80}Fe_{20}$ thin film. *Phys. Rev. B* **56,** 362-366 (1997).

17. Gil, W., Görlitz, D., Horisberger, M. & Kötzler, J. Magnetoresistance anisotropy of polycrystalline cobalt films: Geometrical-size and domain effects. *Phys. Rev. B* **72,** 134401 (2005).

18. Zhang, W., *et al.* Spin Hall Effects in Metallic Antiferromagnets. *Phys. Rev. Lett.* **113,** 196602 (2014).

19. Mendes, J. B. S., *et al.* Large inverse spin Hall effect in the antiferromagnetic metal $Ir_{20}Mn_{80}$. *Phys. Rev. B* **89,** 140406(R) (2014).

20. Wang, X., Vanderbilt, D., Yates, J. R. & Souza, I. Fermi-surface calculation of the anomalous Hall conductivity. *Phys. Rev. B* **76,** 195109 (2007).

21. Yue, D. & Jin, X. Towards a Better Understanding of the Anomalous Hall Effect. *J. Phys. Soc. Jpn.* **86,** 011006 (2017).

22. Zhang, Y. Q., *et al.* Anomalous Hall effect in epitaxial permalloy thin films. *J. Appl. Phys.* **114,** 163714 (2013).

23. Kim, J., *et al.* Spin Hall magnetoresistance in metallic bilayers. *Phys. Rev. Lett.* **116,** 097201 (2016).

24. Naito, T., Hirashima, D. S. & Kontani, H. Tight-binding study of anomalous Hall effect in ferromagnetic 3d transition metals. *Phys. Rev. B* **81,** 195111 (2010).

25. Zimmermann, B., *et al.* Skew scattering in dilute ferromagnetic alloys. *Phys. Rev. B* **90,** 220403(R) (2014).

26. Taniguchi, T., Grollier, J. & Stiles, M. D. Spin-Transfer Torques Generated by the Anomalous Hall Effect and Anisotropic Magnetoresistance. *Phys. Rev. Applied* **3,** 044001 (2015).

27. Taniguchi, T. Magnetoresistance generated from charge-spin conversion by anomalous Hall effect in metallic ferromagnetic/nonmagnetic bilayers. *Phys. Rev. B* **94,** 174440 (2016).

28. Tay, M., Li, K. & Wu, Y. Electrical transport properties of ultrathin metallic films. *J. Vacuum Sci. Technol. B* **23,** 1412 (2005).
